# *Plpp3*, a novel regulator of pluripotency exit and endodermal differentiation of mouse embryonic stem cells

**DOI:** 10.1242/bio.059665

**Published:** 2023-01-12

**Authors:** Martha E. Montané-Romero, Ana V. Martínez-Silva, Augusto C. Poot-Hernández, Diana Escalante-Alcalde

**Affiliations:** ^1^ Instituto de Fisiología Celular, División de Neurociencias, Universidad Nacional Autónoma de México, Ciudad de México C.P. 04510, México; ^2^ Unidad de Bioinformática y Manejo de la Información, Universidad Nacional Autónoma de México, Ciudad de México C.P. 04510, México

**Keywords:** Phospholipid phosphatase type 3, Bioactive lipids, Lysophosphatidic acid, Sphingosine-1-phosphate, Ceramide-1-phosphate, Phosphatidic acid, YAP1, HIPPO, Mouse embryonic stem cells, Pluripotency, Embryoid bodies, Endoderm differentiation

## Abstract

In recent decades, study of the actions of bioactive lipids such as lysophosphatidic acid (LPA) and sphingosine-1-phosphate (S1P) has increased since they are involved in regulating many processes, including self-renewal of embryonic stem cells, embryo development and cancer. Phospholipid phosphatase type 3 (PLPP3) has been shown to be a key player in regulating the balance of these lipids and, in consequence, their signaling. Different lines of evidence suggest that PLPP3 could play a role in endoderm development. To approach this hypothesis, we used mouse embryonic stem cells (ESC) as a model to study *Plpp3* function in self-renewal and the transition towards differentiation. We found that lack of PLPP3 mainly affects endoderm formation during differentiation of suspension-formed embryoid bodies. PLPP3-deficient ESC strongly decrease the amount of FOXA2-expressing cells and fail to properly downregulate the expression of pluripotency factors when subjected to an endoderm-directed differentiation protocol. Impaired endoderm differentiation correlated with a transient reduction in nuclear localization of YAP1. These phenotypes were rescued by transiently restoring the expression of catalytically active hPLPP3. In conclusion, PLPP3 plays a role in downregulating pluripotency-associated factors and in endodermal differentiation. PLPP3 regulates proper lipid/YAP1 signaling required for endodermal differentiation.

## Teaser

**Summary:** We discuss two novel roles of phospholipid phosphatase 3 (PLPP3), in pluripotency exit in mouse embryonic stem cells and in endodermal differentiation through the regulation of bioactive lipid signaling.

## INTRODUCTION

In the past four decades, it became evident that bioactive lipids generated from membrane phospholipids are potent mediators of a wide variety of biological processes. Within the plethora of identified bioactive lipids, lysophosphatidic (LPA) and sphingosine-1-phosphate (S1P) stand out as potent regulators of processes such as cell migration, proliferation, survival, differentiation, inflammation, pain, cancer, genetic and epigenetic transcriptional regulation, amongst others, through their G-protein-coupled receptors or intracellular actions (Engelbrecht et al., 2021; Hannun and Obeid, 2018; Kok et al., 2012; Lidgerwood et al., 2018).

The role of the aforementioned lysophospholipids during vertebrate development has also been actively studied in recent years. Zebrafish with zygotic or maternal-zygotic inactivation of most S1P receptors show no gross developmental defects, with the exception of *s1pr2*, indicating a wide functional redundancy between them (Hisano et al., 2015b). S1P/S1pr2-mediated signaling is required for proper heart development (Fukui et al., 2014; Kupperman et al., 2000), a process in which LPA/Lpar1 seems to play a regulatory function through an antagonistic activity on S1P signaling (Nakanaga et al., 2014). Notably, maternal-zygotic inactivation of *sphk2*, one of the two S1P-synthesizing enzymes, produces a delay in epiboly and phenocopies the *cardia bifida* phenotype observed in *s1pr2* mutants, indicating the key role of S1P in regulating zebrafish heart development (Hisano et al., 2015a; Matsui et al., 2007; Mendelson et al., 2017). In this species, LPA receptors also appear highly redundant, although knockdown studies of single or multiple receptors indicate the participation of Lpar1 in lymphatic vessel development and the synergistic action of Lpar1/Lpar4/Lpar6 in vascular development (Moolenaar et al., 2013; Yukiura et al., 2011). Knockdown or chemical inhibition of Enpp2, the main LPA-synthesizing enzyme [also known as autotaxin (Atx)], produce vascular perturbations and defective formation of the Kupffer's vesicle, a node-like structure involved in left-right patterning, (Frisca et al., 2016; Lai et al., 2012). LPA/Lpar2-mediated signaling is required for proper neural crest migration in *Xenopus* (Kuriyama et al., 2014).

In mice, LPA and S1P receptor redundancy is also indicated by the absence of severe phenotypes before mid-gestation in knockouts (KO) of receptor genes expressed during embryo development. Embryonic phenotypes observed in some single or compound receptor mutants are mainly associated with vascular perturbations, with lethality observed between embryonic day (E)11.5 and E14.5 (for a recent review, see Engelbrecht et al., 2021). Evidence of the relevance of LPA and S1P signaling in early mouse development comes from the gene inactivation or overexpression of lipid-synthesizing enzymes. Mice with overexpression or targeted inactivation of *Ennp2* show embryonic lethality around E10 due to vascular defects, growth retardation and neural tube abnormalities (Koike et al., 2010; van Meeteren et al., 2006). Additionally, *Ennp2* KO embryos show alterations in the formation of large lysosomes of yolk sac visceral endodermal cells (Koike et al., 2009). *Sphk1/2* double KO embryos also show vascular development abnormalities and lethality between E10 and E13, and a subset of them additionally display anterior neural tube closure defect due to increased apoptosis and reduced proliferation of the neuroepithelium (Mizugishi et al., 2005).

Besides their described roles in embryo development, LPA and S1P regulate several aspects of murine and human embryonic, pluripotent and adult stem cells stemness, ranging from self-renewal to moving through different pluripotency states (Garcia-Gonzalo and Izpisua Belmonte, 2008; Kime et al., 2016; Lidgerwood et al., 2018; Pebay et al., 2007; Pitson and Pebay, 2009; Xu et al., 2021).

A group of enzymes that regulate LPA and S1P availability and, in consequence, their signaling are the phospholipid phosphatases 1-3 (PLPP1-3, also known as LPP1-3 or PAP2A-C). These PLPPs are integral membrane enzymes localized on the plasma membrane or the membrane of intracellular organelles (i.e. endoplasmic reticulum, Golgi, endosomes), with their active site looking towards the extracellular space (ecto-activity) or the lumen of organelles. In addition to the hydrolysis of LPA and S1P, these PLPPs also dephosphorylate phosphatidic acid (PA) and ceramide-1-phosphate (C1P), among others (Kok et al., 2012).

Participation of this kind of lipid phosphatases in embryo development was initially described in *Drosophila* (Starz-Gaiano et al., 2001; Zhang et al., 1997), where *Wunen* and *Wunen2* have been shown to be involved in primordial germ cell migration guidance and survival, heart, muscle and trachea development, and *lazaro* in phototransduction (for a recent review, see Lehmann, 2021). In mice, out of the described PLPPs, only the targeted inactivation of *Plpp3* (also known as *Lpp3* or *Ppap2b*) results in embryo lethality before E10.5, mainly due to defects in the vascular development of extraembryonic structures. Conditional inactivation of *Plpp3* in endothelial cells is also lethal between E8.5 and E13, indicating a central role for PLPP3 in vascular development (Chatterjee et al., 2016; Panchatcharam et al., 2014). A subset of *Plpp3*^−/−^ embryos (around 30%) between E6.5 and E9.5 showed severe patterning defects such as accumulation of visceral endoderm (VE) cells at the distal tip, defective anterior visceral endoderm formation (AVE), constrictions at the anterior boundary between embryonic and extraembryonic domains, anterior truncations, duplication of axial structures, demonstrating the participation of PLPP3 in embryo patterning likely associated with defects in VE development (Escalante-Alcalde et al., 2003).

Targeted inactivation of *Plpp3* produces concentration imbalance of substrates and products in the expected sense in a context-dependent fashion (i.e. primary mouse embryo fibroblasts, embryoid bodies, cerebellum, smooth muscle, thymus, spleen, adipose tissue and plasma of mice with conditional inactivation of *Plpp3* in liver or globally reduced after birth), indicating that it plays an important role in regulating the actions of these bioactive lipids, extra- and intra-cellularly (Breart et al., 2011; Busnelli et al., 2017; Escalante-Alcalde et al., 2003; Lopez-Juarez et al., 2011; Mueller et al., 2019; Ramos-Perez et al., 2015; Sanchez-Sanchez et al., 2012). A new role for PLPP3 in S1P cellular uptake/recycling has recently been demonstrated in HeLa cells (Kono et al., 2022). Human and rodent PLPP3 has a functional integrin-binding motif that confers adhesion properties and outside-in signaling when interacting with α_5_β_1_- and α_v_β_3_-expressing cells (Humtsoe et al., 2005, 2003). This domain has been shown to be relevant for endothelial cell-to-cell adhesion and migration *in vitro*; however, the specific role of this domain *in vivo* remains to be elucidated.

In this work, we used *Plpp3*-deficient embryonic stem cells (ESC) as a model to gain further insights into the role of PLPP3 in stemness and during early mouse embryo development. We unveil a critical role for the enzyme in pluripotency exit and in endoderm differentiation, the latter mediated through its activity in regulating lipid-mediated signaling.

## RESULTS

### PLPP3 is not required for the maintenance of pluripotency factors expression in ESC

**Fig. 1.**
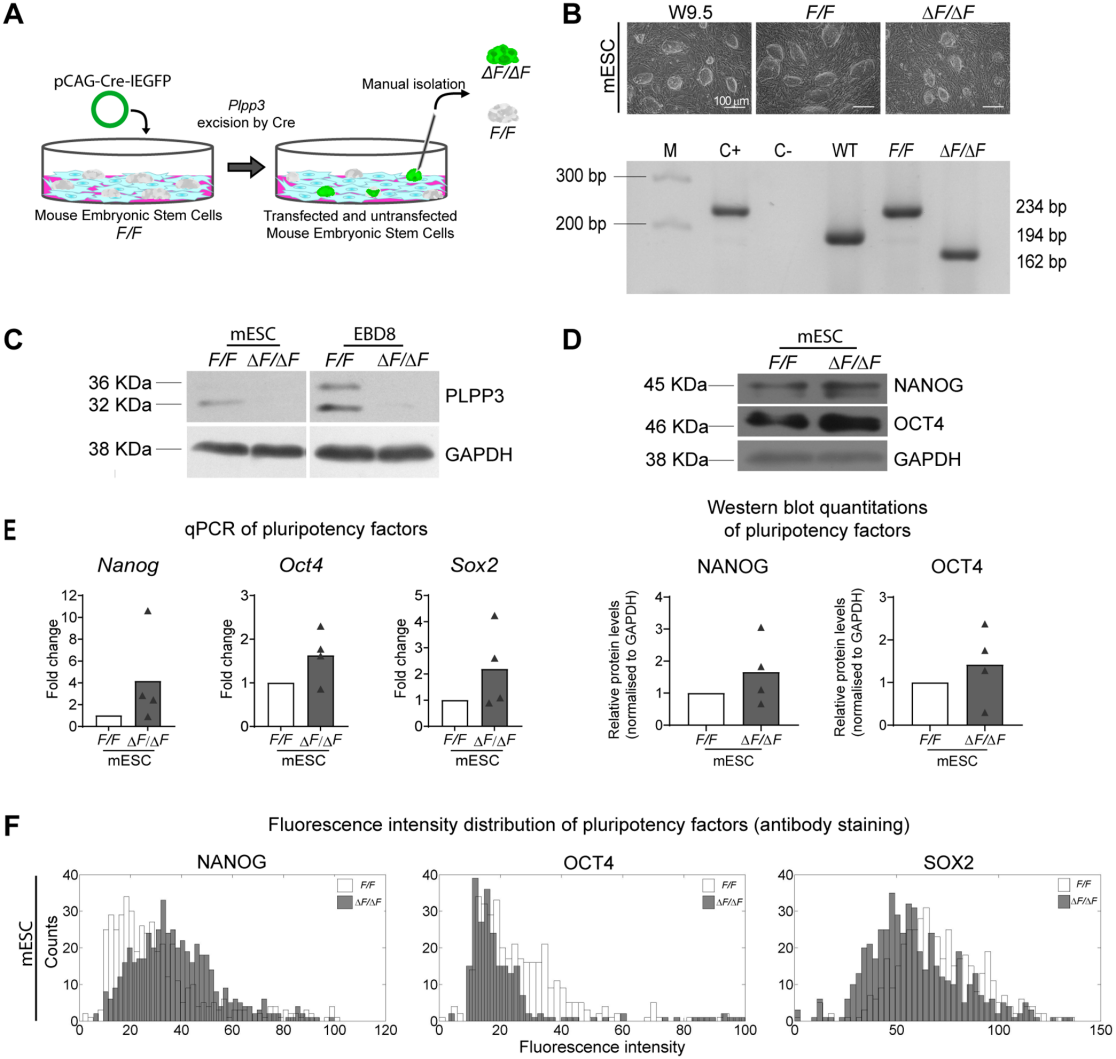
**Lack of PLPP3 does not alter pluripotency factors expression in embryonic stem cells. (**A) Scheme of ESC derivation. (B) (Top) Images of colony morphology. (Bottom) Confirmation of genotypes by PCR of genomic DNA. M, molecular weight marker; C+, *F/F* positive control tail biopsy, C−, negative control; WT, W9.5 wild-type ESC; *F/F*, ESC homozygous for the conditional allele; Δ*F/*Δ*F*, mESC homozygous for the deleted allele. WT product, 194 bp; *F/F* product, 234 bp; Δ*F/*Δ*F* product, 162 bp. (C) Western blots of PLPP3 and GAPDH in *F/F* and Δ*F/*Δ*F* ESC (*n*=1) and *F/F* and Δ*F/*Δ*F* EB differentiated for 8 days (EBD8; *n*=3). Note the lack of protein in mutant cells. The faint band in EB corresponds to unspecific binding. The two bands observed only in *F/F* cells correspond to glycosylated and unglycosylated PLPP3. (D) (Top) Western blot of NANOG, OCT4 and GAPDH in *F/F* and Δ*F/*Δ*F* ESC. (Bottom) Normalized NANOG and OCT4 protein expression in *F/F* and Δ*F/*Δ*F* mESC (*n*=4). (E) Expression of pluripotency factors *Nanog*, *Oct4* and *Sox2* by qPCR in Δ*F/*Δ*F* ESC, expressed as fold change with respect to *F/F* (*n*=4). Comparisons between *F/F* and Δ*F/*Δ*F* were made using two-tailed Mann–Whitney test. (F) Representative histograms showing distribution of fluorescence intensities of nuclei immunostained against NANOG, OCT4 and SOX2 in *F/F* and Δ*F/*Δ*F* ESC (*n*=3). *n*, number of independent experiments.

Mouse embryonic stem cells (mESC) were first established from a *Plpp3^fl/fl^* conditional mouse line with exons 3 and 4 flanked by *loxP* sites (*F/F*) (Fig. 1A,B). This particular allele allows the excision of exons containing a domain essential for its catalytic activity and the integrin-binding domain (Escalante-Alcalde et al., 2007). Pluripotency of derived *F/F* ESC was corroborated by the expression of *Oct4*, *Nanog* and *Sox2* by quantitative PCR (qPCR) and immunofluorescence compared to the known pluripotent W9.5 ESC line (Fig. S1A,C,D) (Escalante-Alcalde et al., 2003; Szabo and Mann, 1994). To evaluate if the lack of PLPP3 affects pluripotency, we generated PLPP3-deficient ESC lines (Δ*F/*Δ*F*) through the transient expression of a bicistronic plasmid expressing CRE recombinase and GFP in *F/F* ESC. GFP^+^ colonies were selected and evaluated for homozygous excision of exons 3 and 4 (Δ*F/*Δ*F*) as described in the Materials and Methods, while GFP^–^ colonies were selected as controls (*F/F*) (Fig. 1A,B). Out of the selected clones, only those with normal karyotype were selected for subsequent experiments. To corroborate the lack of intact PLPP3 protein in Δ*F/*Δ*F* ESC, protein extracts were obtained from feeder-free ESC cultures and derived embryoid bodies (EB) on day (D)8 to rule out the presence of residual wild-type feeders' DNA (Fig. 1C; Fig. S1B).

We next analyzed the expression of pluripotency-associated factors in Δ*F/*Δ*F* and control cell lines, cultured under regular pluripotent conditions, by qPCR and immunofluorescence and found that expression in both cell lines was similar. Also, no significant difference in NANOG and OCT4 expression was observed between cell lines by western blotting (Fig. 1D-F; Fig. S1E). These data indicate that the absence of PLPP3 is compatible with the maintenance of the pluripotent state in murine ESC.

### EB lacking PLPP3 fail to form endoderm cells and to downregulate pluripotency factors

**Fig. 2.**
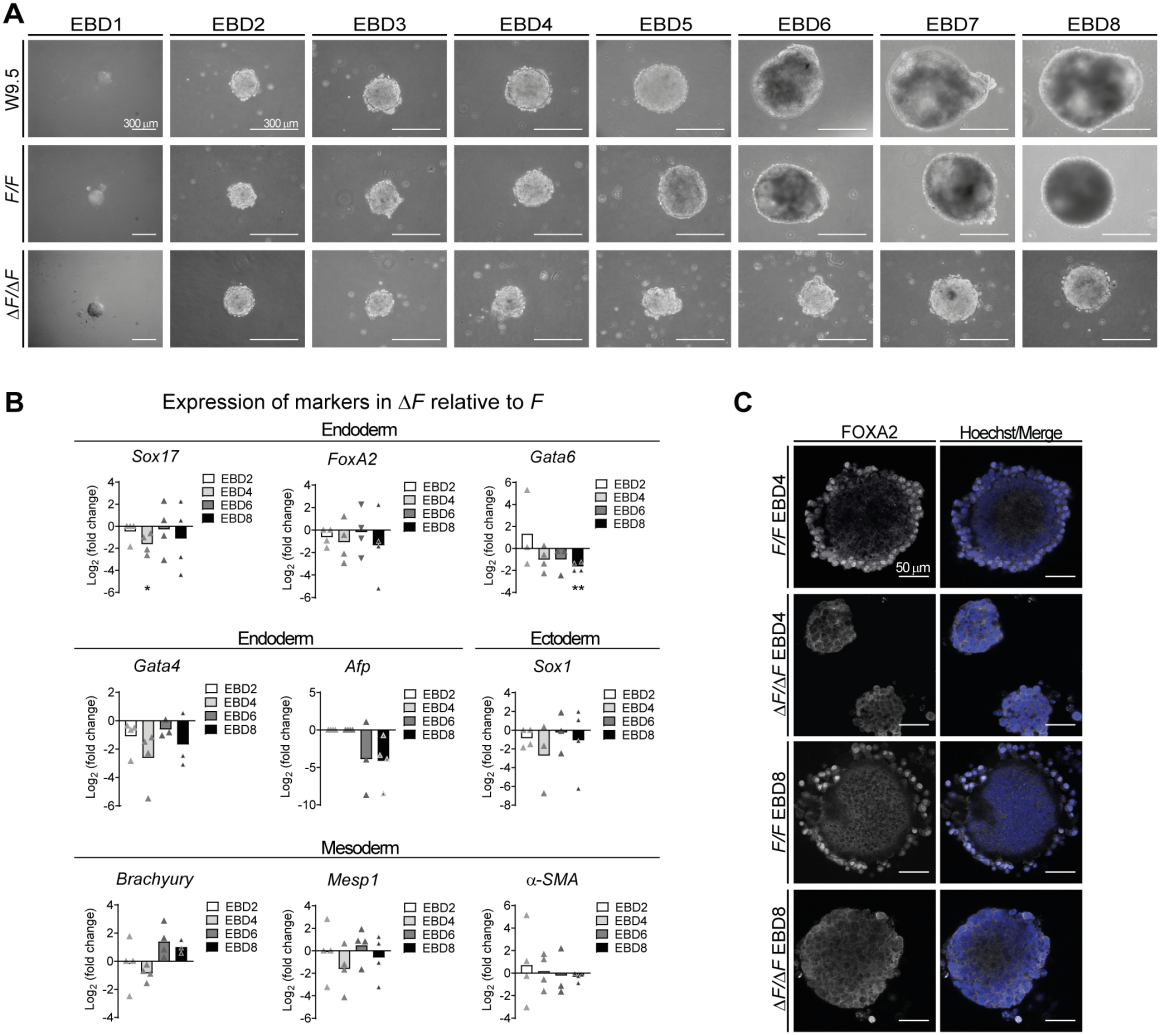
**ESC lacking PLPP3 fail to differentiate endoderm in embryoid bodies.** (A) Hanging drop EB development from D1 up to D8. (B) Expression of lineage-specific transcription factors by qPCR in EB (bulk) at different differentiation time points (2, 4, 6 and 8 days). Graphs represent expression in Δ*F/*Δ*F* expressed as Log_2_ (fold change) with respect to *F/F* (*n*=4); comparisons were made using the Kruskal–Wallis test. (C) *F/F* and Δ*F/*Δ*F* EB immunostained against FOXA2 on D4 and D8. Note the lack of FOX2A^+^ nuclei outer layer in Δ*F/*Δ*F* EB (*n*=3). *P<0.05, **P<0.01. *n*, number of independent experiments.

**Fig. 3.**
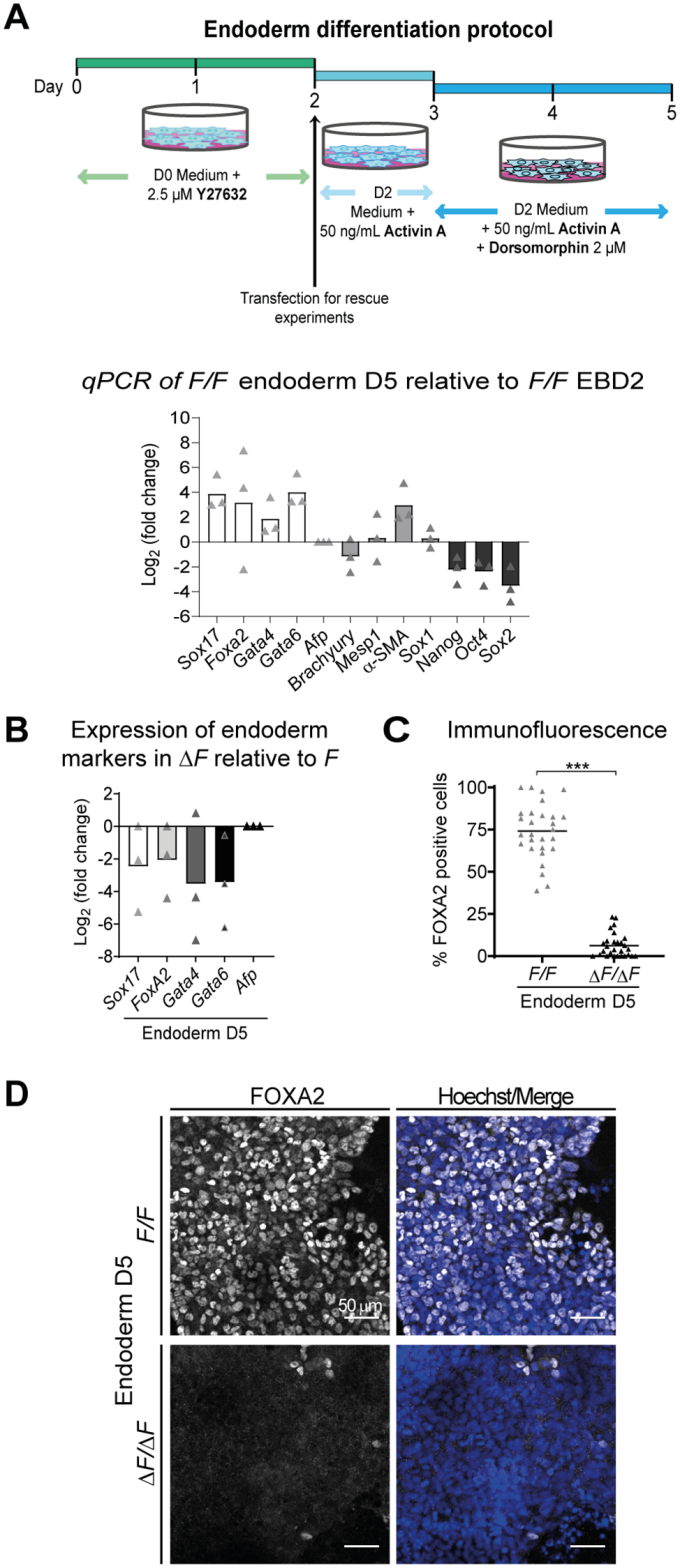
**ESC lacking PLPP3 fail to differentiate to endoderm using a directed monolayer differentiation protocol.** (A) (Top) Scheme of the endoderm differentiation protocol. (Bottom) Expression of pluripotency and germ layer differentiation markers in differentiated *F/F* cell to endoderm on D5. Data are expressed as Log_2_ (fold change) with respect to *F/F* EB on D2 (*n*=3). (B) Expression of endoderm differentiation markers by qPCR in ESC differentiated towards endoderm (D5). Expression in Δ*F/*Δ*F* is expressed as Log_2_ (fold change) with respect to *F/F* cells (*n*=3). (C) Percentage of FOXA2^+^ nuclei in *F/F* and Δ*F/*Δ*F* cells differentiated to endoderm (*n*=4). (D) FOXA2 immunodetection in *F/F* and Δ*F/*Δ*F* ESC differentiated to endoderm (*n*=4). *n*, number of independent experiments. Comparisons between *F/F* and Δ*F/*Δ*F* were made using two-tailed Mann–Whitney test. ***P<0.001.

To analyze the role of PLPP3 during differentiation, we established EB from ESC. We used the hanging drop method for homogeneous formation of EB, and growth differences were evaluated. EB formed from control ESC lines increased in size as differentiation days passed; the peripheral layer of endoderm-like epithelial cells was evident around D4, and a proportion of EB cavitated from D6 onwards. In contrast, EB formed from Δ*F/*Δ*F* ESC were evidently smaller by D4, and the outer layer of endoderm epithelial cells and cavitation were not observed (Fig. 2A). VE dysfunction generates cavitation and differentiation defects in EB (Brickman and Serup, 2017; Coucouvanis and Martin, 1999; Esner et al., 2002); therefore, we then examined the kinetics of expression of germ layer differentiation markers by qPCR in EB differentiated in static suspension culture between D2 and D8 (Fig. 2B). In the absence of PLPP3, expression of all endoderm differentiation markers had a trend to decrease with respect to control EB in the majority of time points analyzed, with expression of *Sox17* showing significant and consistent reduction on D4. To corroborate whether PLPP3 deficiency affects this lineage during EB differentiation and due to the variability found in qPCR experiments, we subjected D4 and D8 EB to immunofluorescence of the visceral and definitive endoderm marker FOXA2 (E5.5-E7.5) (Burtscher and Lickert, 2009; Perea-Gomez et al., 1999). While cells with FOXA2^+^ nuclei were observed surrounding the periphery of D4 and D8 control EB, no positive cells for this marker were observed in Δ*F/*Δ*F* EB at the same time points (Fig. 2C), suggesting an endodermal dysfunction. To establish whether this effect was due to failure in differentiation or detachment of endodermal cells from EB, we subjected ESC to an activin-driven endoderm monolayer differentiation protocol and differentiation corroborated by expression of germ layer markers by qPCR (Fig. 3A). Differentiation markers were analyzed by qPCR and immunofluorescence at D5. Expression of endoderm markers assayed (except for *Afp*, which was not expressed in cells of both genotypes) was reduced in Δ*F/*Δ*F* cells with respect to *F/F* controls (Fig. 3B), and the proportion of cells with nuclear FOX2A was extremely reduced: 7% in Δ*F/*Δ*F* versus 74% in *F/F* (Fig. 3C,D).

**Fig. 4.**
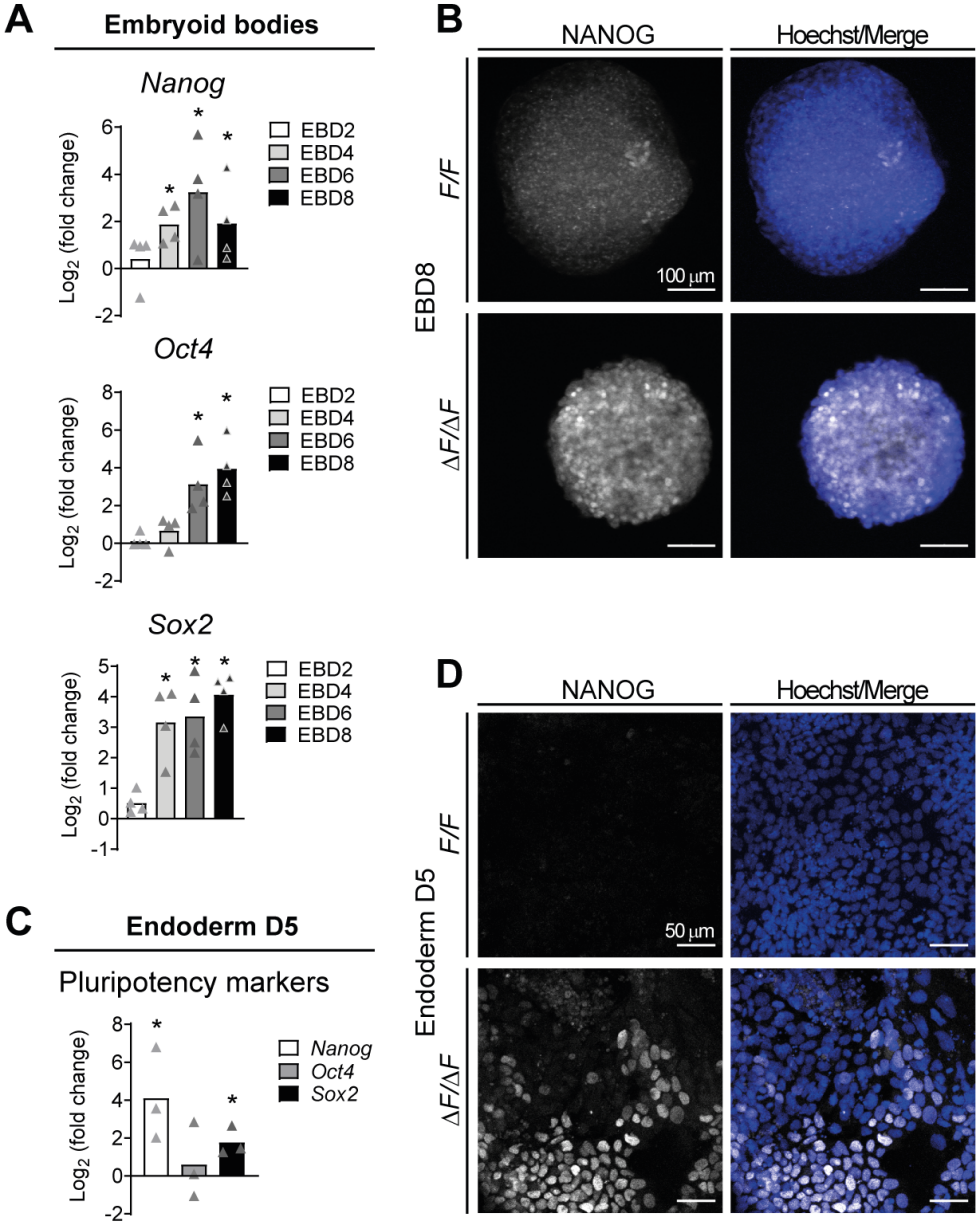
**Δ*F/*Δ*F* cells fail to downregulate pluripotency factors during differentiation.** (A) Expression of pluripotency transcription factors *Nanog*, *Oct4* and *Sox2* in EB at different differentiation time points. Expression in Δ*F/*Δ*F* cells is shown as Log_2_ (fold change) with respect to *F/F* cells (*n*=4). (B) NANOG immunostaining in *F/F* and Δ*F/*Δ*F* EBD8. (C) Expression of pluripotency transcription factors *Nanog*, *Oct4* and *Sox2* expressed as Log_2_ (fold change) with respect to *F/F* cells in cells differentiated towards endoderm on D5 (*n*=3). (D) NANOG immunostaining in *F/F* and Δ*F/*Δ*F* ESC differentiated towards endoderm on D5. *n*, number of independent experiments. Comparisons between *F/F* and Δ*F/*Δ*F* were made using two-tailed Mann–Whitney test; for multiple group comparisons, the Kruskal–Wallis test was used. *P<0.05.

As part of the analysis of lineage-specific markers in EB or endoderm-differentiated cultures, we also analyzed the expression kinetics of the pluripotency markers *Nanog*, *Oct4* and *Sox2*. Surprisingly, we did not observe the expected gradual downregulation of these factors' mRNA as the differentiation progressed in either EB or differentiated monolayers lacking PLPP3 (Fig. 4A,C). To corroborate this observation, we performed immunofluorescence against NANOG in EB and differentiated monolayers. Remarkably, while a very low proportion of cells still expressed NANOG in *F/F* EB at D8 and differentiated monolayers at D5, in those derived from Δ*F/*Δ*F* cells a very high proportion of NANOG^+^ cells was still present, indicating that PLPP3 somehow participates in repressing NANOG expression throughout ESC differentiation (Fig. 4B,D). These data indicate that PLPP3 is required for endoderm differentiation and that it allows pluripotency exit during ESC differentiation.

### YAP1 nucleus-cytoplasmic distribution is transiently altered in Δ*F/*Δ*F* cells differentiated to endoderm

The extracellular concentration of LPA and S1P, two PLPP3 substrates, would be affected by the enzyme's deficiency potentially altering signaling pathway(s) linked to endoderm differentiation or maintenance of pluripotency (MAPK/ERK1/2, AKT, WNT/β-CATENIN, ACTIVIN/SMAD2, JAK/STAT3 and HIPPO). We analyzed the activity of several of these pathways by western blotting in feeder-free ESC EB at different time points during differentiation, as well as cells differentiated to endoderm. In ESC, we did not find significant differences in ERK, AKT, β-CATENIN or STAT3 activation in Δ*F/*Δ*F* with respect to *F/F* cells, although a great variability was observed in active AKT in mutant cells (Fig. S2A). During EB differentiation, no significant differences in active ERK, AKT, β-CATENIN or SMAD2 were observed between *F/F* and Δ*F/*Δ*F*, although a great variability was observed for SMAD2 on D8 and for β-CATENIN on D4-D8 in Δ*F/*Δ*F* EB (Fig. S2C-F).

In cells differentiated towards endoderm, no significant differences in active ERK, AKT, β-CATENIN or SMAD2 were observed between genotypes, but a great variability in activity was observed for ERK and β-CATENIN in Δ*F/*Δ*F* cells (Fig. S2B). The lack of significant functional differences was confirmed by immunofluorescence and analysis of nuclear localization of active β-CATENIN and p-ERK (Fig. S4A,C; data not shown).

In mESC, YAP1, the transcriptional co-activator of the HIPPO pathway, is dispensable for self-renewal but is critical for their differentiation (Chung et al., 2016). LPA and S1P can activate or inhibit the HIPPO-YAP1 pathway depending on the coupled G-protein activated by their receptors (Yu et al., 2012), and these bioactive lipids have been involved in regulating self-renewal in ESC. Based on this, we explored possible alterations to the HIPPO-YAP1 pathway in PLPP3-deficient ESC differentiated to endoderm.

**Fig. 5.**
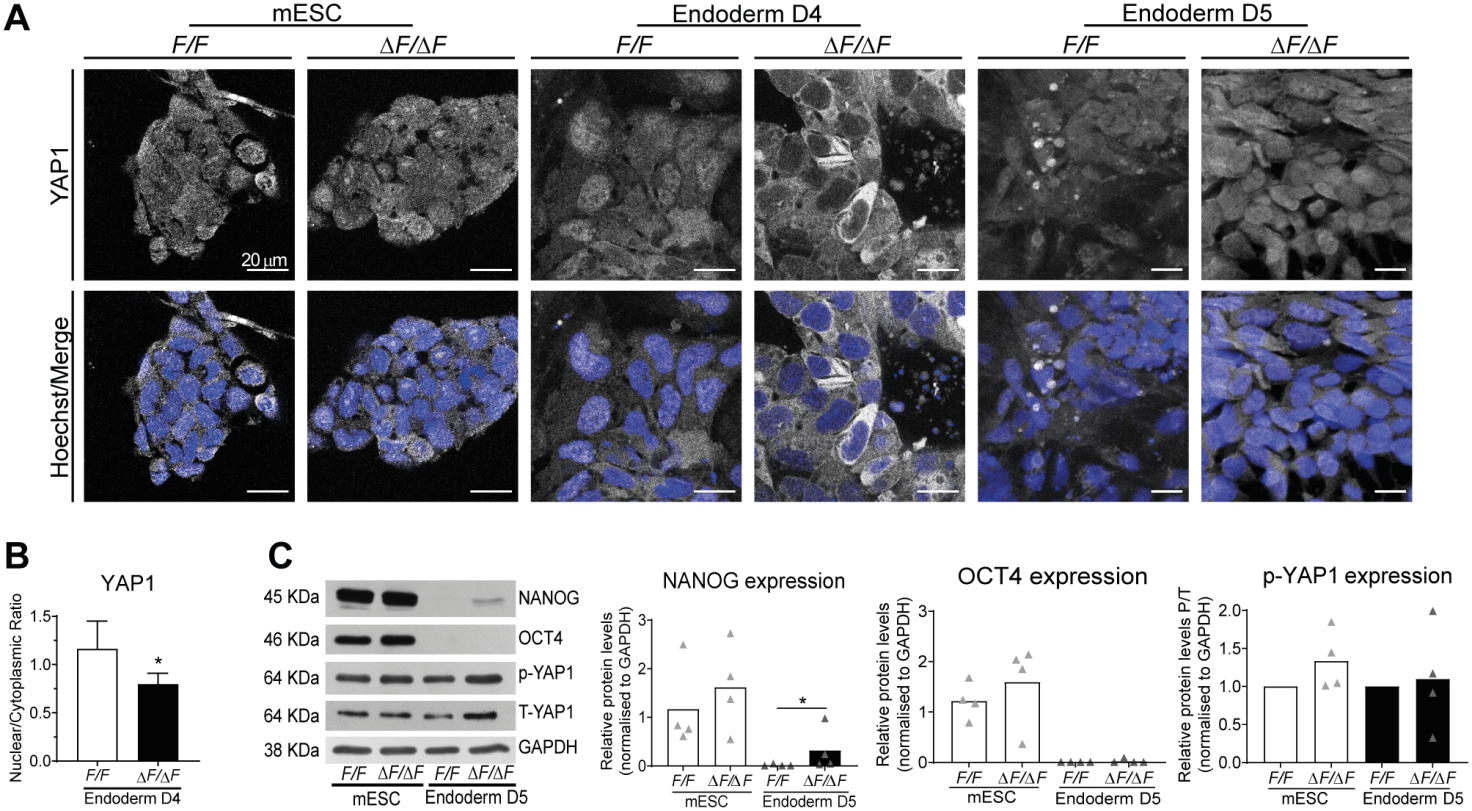
**YAP1 nuclear localization is transiently altered in Δ*F/*Δ*F* cells differentiating to endoderm. (**A) YAP1 immunostaining performed on *F/F* and Δ*F/*Δ*F* ESC and cells differentiated to endoderm at D4 and D5. Note the drastic reduction in nuclear YAP1 in Δ*F/*Δ*F* cells differentiated for 4 days with respect to control cells. (B) Bar plot showing nuclear/cytoplasmic ratio of YAP1 in *F/F* and Δ*F/*Δ*F* endoderm-differentiating cells at D4 (*n*=5), Data are shown as mean±s.d. Comparisons between *F/F* and Δ*F/*Δ*F* nuclear/cytoplasmic ratio were made using two-tailed Student's *t*-test. (C) (Left) Western blot of NANOG, OCT4, pYAP1, total YAP1 and GAPDH in *F/F* and Δ*F/*Δ*F* ESC and endoderm differentiated cells on D5. (Right) Bar plots showing normalized protein levels (*n*=4). Comparisons were made using two-tailed Mann–Whitney test. *P<0.05. *n*, number of independent experiments.

We initially evaluated YAP1 expression and subcellular localization in *F/F* and Δ*F/*Δ*F* ESC and found no difference in content and distribution (Fig. 5A,C). Then, we analyzed YAP1 in ESC differentiating toward endoderm on D4 and D5. Notably on D4, YAP1 was mostly cytoplasmic in Δ*F/*Δ*F* cells, while in *F/F* cells nuclear localization of YAP1 was evident (Fig. 5A). Consistent with the latter observation, a significant decrease in the YAP1 nucleus/cytoplasm ratio was observed in PLPP3-deficient cells (0.8 in Δ*F/*Δ*F* versus 1.2 in *F/F*; Fig. 5B). On D5, we did not observe any evident difference in distribution or expression of YAP1 between genotypes, but persistence of NANOG expression was still detected in PLPP3-deficient cells (Fig. 5A,C). Altogether, our data show that the absence of PLPP3 alters endoderm differentiation and that this correlated with a drastic and transient inactivation of YAP1 (cytoplasmic YAP1) on D4 of differentiation.

### Endoderm differentiation from Δ*F/*Δ*F* ESC is rescued by expression of wild-type hPLPP3 in a non-cell autonomous fashion

As mentioned before, murine and human PLPP3 are bifunctional proteins that, besides phosphatase activity, possess a functional integrin-binding domain that mediates adhesion/aggregation between cells and activates integrin-mediated signaling. To identify the functional domains of PLPP3 responsible for the observed phenotypes, we conducted rescue experiments in differentiating endodermal cells transfected with plasmids bearing different versions of the human enzyme fused to DsRed (Table S4): *DsRed-Plpp3*, wild type; *DsRed-RAD*, lacking the integrin-binding motif; *DsRed-AS*, inactive catalytic site; *DsRed-RAD AS*, integrin-binding and catalytic site double mutant. Transfection of vectors was done on the second day of the differentiation protocol (Fig. 3A) and assayed on D4 or D5 by FOXA2 and NANOG immunostaining and distribution of YAP1.

**Fig. 6.**
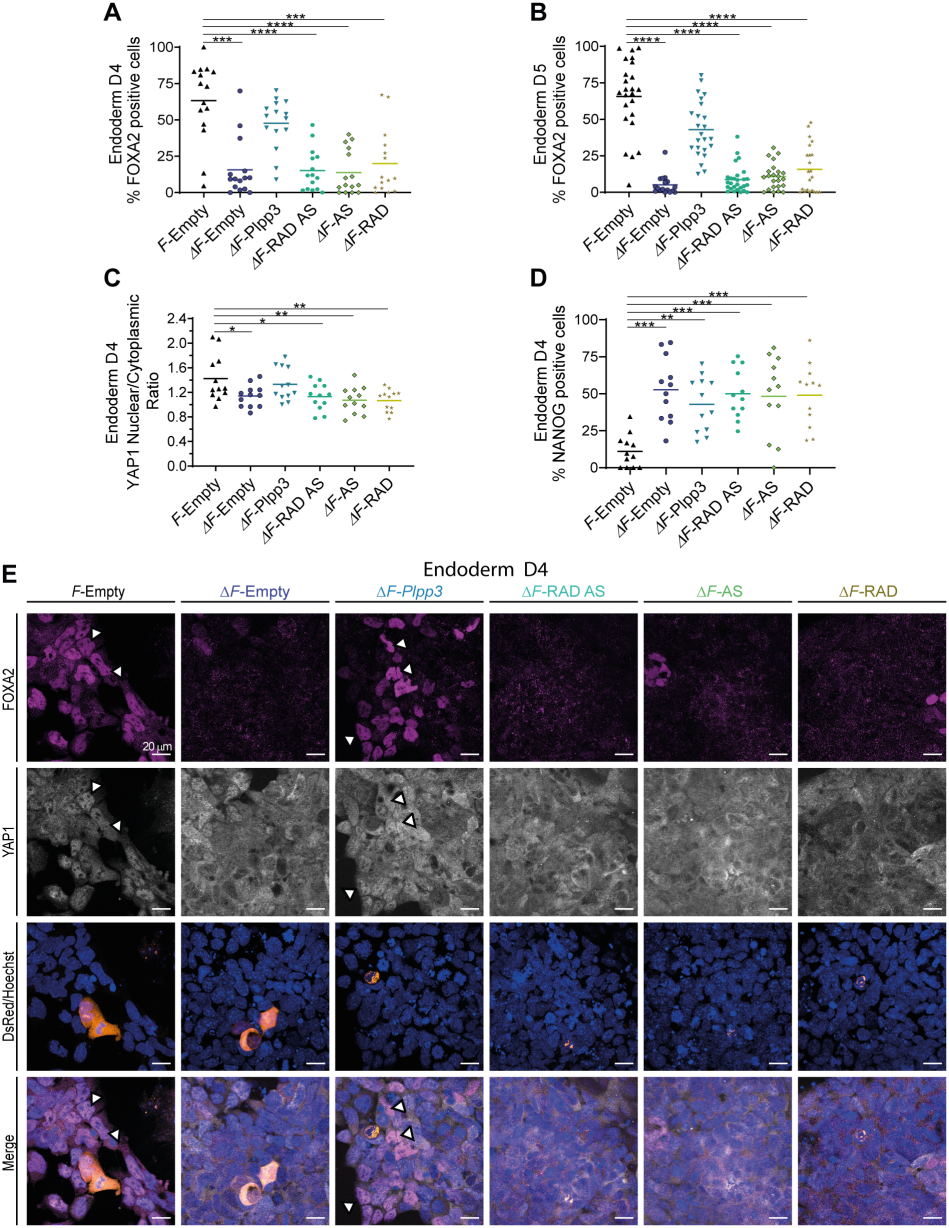
**Rescue experiments in PLPP3-deficient cells differentiating towards endoderm.** (A) Percentage of FOXA2^+^ nuclei in cells differentiated to endoderm at D4, transfected with the indicated vectors (*n*=3). (B) Percentage of FOXA2^+^ nuclei in cells differentiated to endoderm at D5, transfected with the indicated vectors (*n*=3). (C) YAP1 nuclear/cytoplasmic ratio in cells differentiated to endoderm at D4, transfected with the indicated vectors (*n*=3). (D) Percentage of NANOG^+^ nuclei in cells differentiated to endoderm at D4, transfected with the indicated vectors. Phenotypic rescue does not require PLPP3 being expressed in rescued cells, suggesting a non-cell-autonomous effect (*n*=3). (E) Representative experiment. Immunofluorescence against FOXA2 and YAP1 in endoderm-differentiating cells at D4, transfected with the indicated *DsRed* vectors. In *F/F* cells transfected with the empty vector, the majority of FOXA2^+^ nuclei also have nuclear YAP1 (white arrowheads), while in Δ*F/*Δ*F* cells transfected with the empty vector the majority of the cells are FOXA2^−^ and show strong YAP1 cytoplasmic staining. The phenotype can be reverted by transfecting Δ*F/*Δ*F* cells with the WT version of the enzyme (white arrowheads) but not by transfection with single-domain or double-domain mutants. *F*, *F/F* cells; Δ*F*, Δ*F/*Δ*F* cells; Empty, *DsRed*-empty vector; *Plpp3*, *DsRed*-wild-type *Plpp3*; RAD, *DsRed-Plpp3* lacking the integrin-binding motif; AS, *DsRed-Plpp3* with inactive catalytic site; RAD AS, *DsRed-Plpp3* integrin-binding motif and catalytic site double mutant. *n*, number of independent experiments; each point in the dot plots corresponds to one analyzed photo. Comparisons were made with respect to *F*-Empty using the Kruskal–Wallis test, except for YAP nuclear/cytoplasmic ratio, where comparisons were made using one-way ANOVA. *P<0.05, **P<0.01, ***P<0.001, ****P<0.0001.

Cells transfected with wild-type hPLPP3 (*DsRed-Plpp3*) rescued the appearance of FOXA2^+^ nuclei in enzyme-deficient cells on D4; the quantity of cells expressing this transcriptional factor approached that found in *F/F* cells transfected with empty vector (Fig. 6A,E). Similar findings were observed in experiments carried out on D5 (Fig. 6B; Fig. S3). Notably, FOXA2^+^ cells were more frequent in the vicinity of remaining transfected cells (20% transfection efficiency at 24 h), indicating a non-cell-autonomous effect. Similar findings were observed in experiments carried out on D5. Furthermore, we did not observe rescue with any of the mutant versions of PLPP3 (Fig. 6A,E). Coincidentally, Δ*F/*Δ*F*-rescued cells expressing FOXA2 on D4 also showed nuclear localization of YAP1 similar to *F/F* cells; however, cells transfected with any of the mutant variants behave as unrescued cells, with YAP1 mostly cytoplasmic (Fig. 6C,E). These results suggest that YAP1 translocation to the nucleus contributes to endoderm differentiation and requires PLPP3 catalytic activity to produce specific extracellular lipid signaling in this cellular context.

**Fig. 7.**
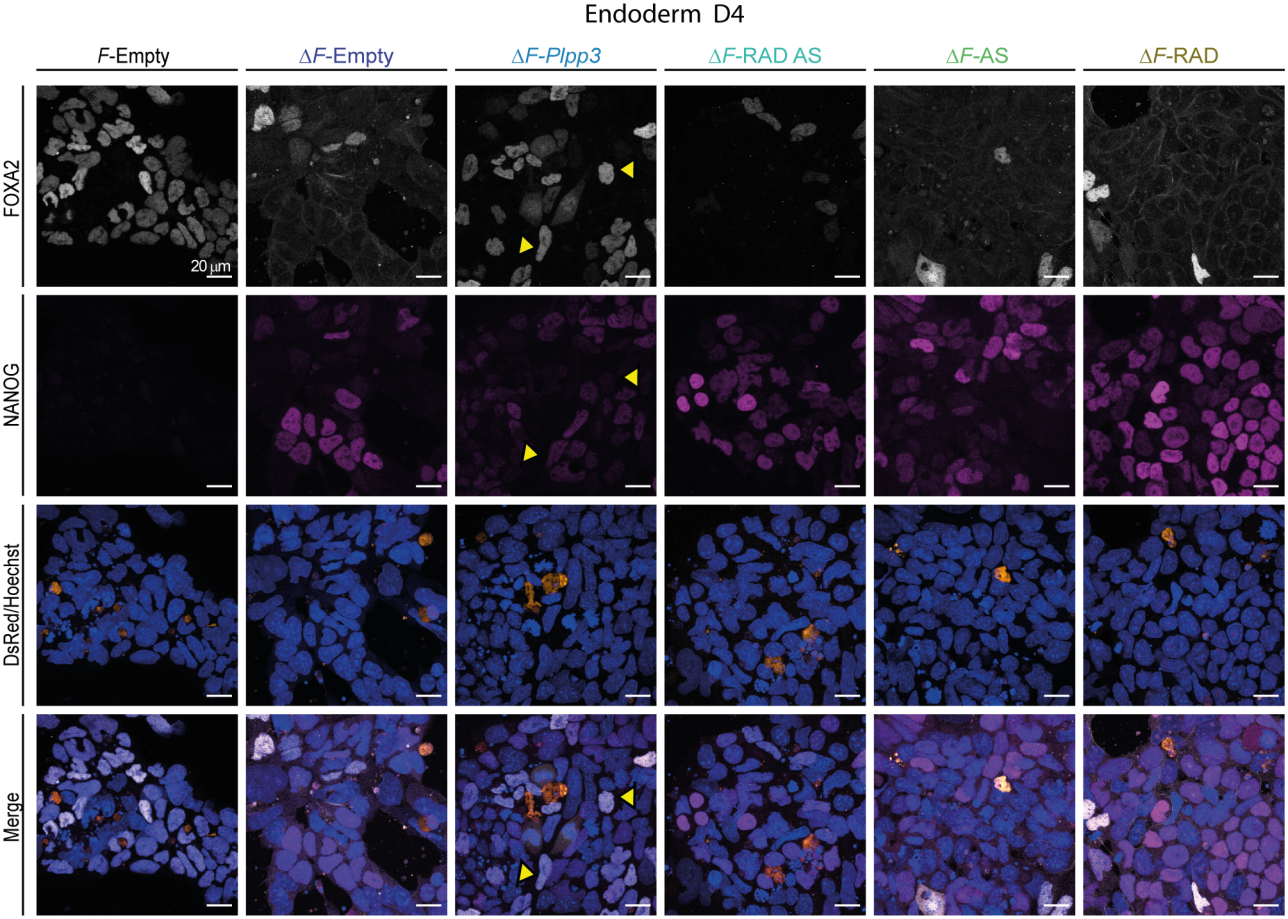
**FOXA2 expression in rescued cells does not correlate with reduction of NANOG-positive cells.** Representative experiment. Immunofluorescence against FOXA2 and NANOG in endoderm-differentiating cells at D4, transfected with the indicated *DsRed* vectors. No reduction in the amount of NANOG^+^ cells was observed in Δ*F/*Δ*F* cells transfected with wild-type, single-domain or double-domain *Plpp3* variants. The majority of FOXA2^+^ nuclei in rescued Δ*F/*Δ*F* cells were NANOG^+^ (yellow arrowheads). *F*, *F/F* cells; Δ*F*, Δ*F/*Δ*F* cells.

Persistence of NANOG expression (58% of positive nuclei) found on ‘differentiated’ cells lacking PLPP3 was not decreased by transfection with the wild-type version of the enzyme nor with any of the mutants (Fig. 6D, Fig. 7; Fig. S4C). Also, we noticed that in all Δ*F/*Δ*F* cells, regardless of the version of *Plpp3* transfected, a larger proportion of cells showing lower and higher NANOG fluorescence intensities than in *F/F* cells was present (Fig. 7; Fig. S4,C). Furthermore, expression of NANOG and FOXA2 in Δ*F/*Δ*F-*rescued cells was mainly mutually exclusive (Fig. 7). These results show that PLPP3 somehow contributes to downregulating NANOG during endodermal differentiation by a mechanism still to be established.

## DISCUSSION

ESC are a powerful tool to study embryo development and to reveal the molecular and cellular processes dysregulated by mutations affecting development. Our previous work pointed to defective VE development in a subset of embryos lacking PLPP3 originating severe anterior-posterior patterning defects. To gain further insight into the relevance of *Plpp3* during early embryonic development, we derived homozygous mutant ESC from conditional *Plpp3^fl/fl^* blastocyst and analyzed their pluripotency status and differentiation potential initially through the formation and analysis of EB.

Lack of PLPP3 did not alter the pluripotency status of ESC when cultured under regular culture conditions [feeders/LIF/15% fetal bovine serum (FBS)]. This observation was not unexpected since LPA and S1P (substrates potentially altered in the PLPP3 mutant) have been shown to have beneficial effects on mESC proliferation and promotion/maintenance of the naïve pluripotent state (Kime et al., 2016; Rodgers et al., 2009; Ryu et al., 2014; Smith et al., 2013; Todorova et al., 2009) through the broad expression of their lipid receptors (Lidgerwood et al., 2018). Paradoxically, we did not detect significant changes in signaling pathways associated with the maintenance of pluripotency in PLPP3-deficient ESC cultures, although we observed great variability in AKT activation, previously described to be able to substitute LIF for the maintenance of pluripotency (Watanabe et al., 2006).

Our work allowed us to unveil defects during PLPP3-deficient EB differentiation, represented by defective formation of the outer endoderm (PrE/VE-like) in hanging drop- and suspension-formed EB, a generalized trend for reduction of the three germ layer differentiation markers, except for some mesodermal markers, for which the trend was to increase at some differentiation time points, and failure to properly downregulate the pluripotency markers *Oct4*, *Nanog* and *Sox2*.

Induction of PrE/VE-like and differentiation of the epiblast-like cells in EB is interdependent. In this sense, several mechanisms could explain our observations in EB: failure to properly exit the pluripotent state affecting PrE/VE differentiation, bias to differentiate to a specific lineage, an autonomous defect in PrE/VE differentiation that affected epiblast differentiation, or a combination of mechanisms.

Evidence of a possible bias to differentiate into mesodermal derivatives in EB comes from the present and previous studies. Here, we observed a strong trend for increased active β-CATENIN in D4 and D6 mutant EB. We previously reported, using a different *Plpp3* allele, the increased and prolonged expression of *Brachyury* in *Plpp3*^−/−^ EB, presumably due to the lack of its antagonistic role in β-CATENIN-mediated TCF transcriptional activity and Xwnt8-mediated *Xenopus* axis duplication assays (Escalante-Alcalde et al., 2003). Furthermore, lack of PLPP3 promoted the differentiation of SMA^+^ cells in ESC subjected to a spinal neuron-directed differentiation protocol (Sanchez-Sanchez et al., 2012).

Despite the reported beneficial effect of S1P and particularly of LPA signaling in proliferation and in promoting/maintaining the naïve pluripotent state of mESC, to our knowledge, no data exist regarding the role of continuously supplementing LPA and/or S1P (besides the lipids present in FBS) during classical EB differentiation, which somehow would equate the effect of lacking PLPP3. Interestingly, the knockdown in mESC of the enzyme that catalyzes the irreversible degradation of intracellular S1P, sphingosine phosphate lyase (*Sgpl*-KD), resulted in a 5-fold increase in intracellular S1P, higher expression of some pluripotency markers (SSEA1 and OCT4) and strong activation of STAT3. Reportedly, *Sgpl*-KD EB were able to form; unfortunately, their characterization was just limited to a marker of stem cells of mesodermal origin (Smith et al., 2013). In this work, we did not observe differences in the activation of STAT3 between control and PLPP3-deficient ESC cultures. On the other hand, LPA-mediated signaling through LPAR1/ROCK, in combination with other factors (LIF, BMP4 and ascorbic acid), converts primed mouse epiblast stem cells into naïve ESC, and LPAR1-mediated signaling seems to positively regulate NANOG expression (Kime et al., 2016). Assuming that *Sgpl*-KD EB differentiate three germ layers normally, this would indicate that LPA- more than S1P-mediated signaling, or a combination of both, would be responsible for the alterations we observed.

In this sense, is tempting to speculate that the lack of downregulation of pluripotency-associated transcription factors would be the result of altered lipid signaling precluding mESC to properly exit pluripotency. This is in line with observations where downregulation of *Nanog* is required for the differentiation of the PrE/VE layer in EB, while its forced expression in mESC has been shown to prevent PrE/VE formation in EB (Hamazaki et al., 2004).

Aiming to sort out if the main target of the PLPP3 deficiency was the endoderm lineage – based on its expression pattern and severe patterning defects of mutants during early post-implantation development – we applied an endoderm-directed differentiation protocol to ESC. Remarkably, endoderm differentiation of Δ*F/*Δ*F* ESC under this condition was also impaired (assessed by qPCR of endoderm markers and FOXA2 immunodetection) concomitant with failure to downregulate pluripotency factors (assessed by qPCR of markers and NANOG immunodetection/western blotting), supporting that the main defect produced by PLPP3 deficiency occurs in the endodermal lineage, either in the VE, definitive endoderm (DE) or both. These data also suggest that PLPP3 deficiency during endoderm differentiation of ESC produces a lipid signaling imbalance (increased signaling or receptor desensitization) and/or an RGE/integrin-mediated alteration that, in turn, would prevent them from exiting the pluripotent state.

Our rescue experiments suggest that the lipid phosphatase ecto-activity of PLPP3 is required for endoderm differentiation since only a small quantity of cells expressing wild-type *hPLPP3* were enough to induce the appearance of FOXA2^+^ cells and nuclear localization of YAP1 in Δ*F/*Δ*F* cells on D4. This indicates that an appropriate LPA and/or S1P signaling produced by PLPP3 is required for proper endoderm development. This also suggests that the *hPLPP3-RAD* mutant also alters the phosphatase catalytic activity, since it was unable produce the same rescue effect seen with wild-type *hPLPP3.*

LPA and S1P signaling crosstalk with the HIPPO-YAP1 pathway has been described (Yu et al., 2012). In mESC, it has previously been shown that the YES-YAP-TEAD2 signaling pathway induces promoter activity of *Oct4* and *Nanog* (Tamm et al., 2011). Some studies suggest that nuclear YAP1 is required for self-renewal downstream of LIF, while others found no impact on stemness or proliferation (LeBlanc et al., 2021). In human pluripotent stem cells (hPSC), YAP1-mediated transcriptional activity, promoted by LPA signaling, generates a naïve-like transcriptional profile and epigenetic changes compatible with this state represented by reduction in the heterochromatic marker H3K9me3 (Qin et al., 2016). The role of this pathway during ESC differentiation is also conflicting. Decreased TEAD2 activity, through a repressor fusion construct, induced endoderm-specific markers (Tamm et al., 2011); overexpression of YES, YAP1 or active-TEAD2 induced differentiation in one study (Tamm et al., 2011) while in another (Lian et al., 2010) *Yap1* overexpression maintains the ESC phenotype even under differentiation conditions; in another study (Chung et al., 2016), *Yap1*-KO ESC showed impaired differentiation while *Yap1*-KD produced loss of pluripotency (Lian et al., 2010).

Despite contradictory studies regarding the role of YAP1 in mESC pluripotency and differentiation, translocation of YAP1 to the nucleus is a key feature of spontaneously differentiated mESC after LIF removal (Chung et al., 2016). Furthermore, its role in VE and early definitive endoderm development is strongly supported by the more severe phenotype of *Yap1*-deficient embryos and spatiotemporal transcriptomics analyses (Morin-Kensicki et al., 2006; Peng et al., 2019). Our data strongly suggest that lipid signaling imbalance caused by the absence of PLPP3 alters YAP1 nuclear localization, leading to defective endoderm differentiation. Although we did not demonstrate a direct link between YAP1 and *Foxa2* transcription, it is worth noting that, in D4 rescued *Plpp3* mutant cells, FOXA2^+^ nuclei also showed nuclear YAP1.

Some phenotypic characteristics are shared between severely affected *Yap1*, *Foxa2* and *Plpp3* KO mouse embryos such as constrictions at the boundary between embryonic and extraembryonic domains, and accumulation of VE cells at the distal tip of the embryo around E7.0 (Ang and Rossant, 1994; Escalante-Alcalde et al., 2003; Morin-Kensicki et al., 2006). These phenotypes could be attributed to VE development abnormalities since they are rescued by wild-type expression of *Foxa2* or *Plpp3* in the VE in wild-type ⇔ mutant chimaeras (Dufort et al., 1998; Escalante-Alcalde et al., 2003).

In zebrafish, S1P/S1pr2/Yap1 signaling regulates endoderm formation and convergence required for cardiac progenitor cell migration. Alterations in these S1P-regulated processes lead to the appearance of *cardia bifida* (Fukui et al., 2014; Ye and Lin, 2013), a phenotype that is enhanced by overexpression of *atx* and is mediated by *lpar1*, indicating an antagonistic role of LPA in the S1P signaling involved in these processes (Nakanaga et al., 2014). Notably, a small proportion of *Plpp3^tm1Stw/tm1Stw^* mouse embryos with severe abnormalities also displayed defects in the ventral closure of the gut endoderm causing *cardia bifida* (Busnelli et al., 2018), suggesting conserved mechanisms.

Single-cell RNA-sequencing (scRNA-seq) analyses of developing mouse embryos (Nowotschin et al., 2019; https://endoderm-explorer.com/) show that *Plpp1/2/3*, LPA- and S1P-synthesizing enzymes (*Enpp2*, *Sphk1/2*), S1P phosphatases (*Sgpp1/2*), S1P lyase (*Sgpl1*) and LPA/S1P receptors (*Lpar/S1pr*) have a dynamic and differential expression between inner cell mass (ICM)/epiblast/epiblast-derived cells and endodermal lineages cells in E3.5-E7.5 mouse embryos (Fig. S5). *Plpp1/2/3* are expressed in the ICM of E3.5 blastocyst and become downregulated in epiblast cells of E4.5 embryos but maintained in the endoderm lineage. In PrE/VE cells between E4.5 and E5.5, *Plpp1* shows a sharp decline in expression while *Plpp2* and *Plpp3* only show a slight reduction, but later their expression increases. Between E6.5 and E7.5, *Plpp2* and *Plpp3* are expressed in the extraembryonic (exVE) and embryonic (emVE) VE; however, *Plpp3* expression is more prominent in the emVE, whereas *Plpp1* maintains low levels of expression in this tissue during this period. *Plpp1/2/3* show detectable expression in nascent definitive endoderm from E7.0 onwards (Fig. S5). These suggest that substrate concentration regulation is key in the ICM (ESC-like state) and then becomes more relevant in PrE/VE lineages than in epiblast cells up to E7.0, when *Plpp1-3* start to show variable expression levels in different epiblast derivatives. Our conclusions are in agreement with these transcriptomic data regarding the timing and lineage-specific requirement of PLPP3. One puzzling question arising from these data is that, despite *Plpp2* and *Plpp3* being expressed simultaneously in the VE, only the inactivation of *Plpp3* produces severe developmental defects. These differences could rest on differential subcellular localization of each enzyme during development or their differential roles in regulating intracellular pools of PA and S1P (Long et al., 2005, 2008).

LPA signaling-associated genes *Enpp2*, *Lpar1*, *Lpar2* and *Lpar6* are the ones with significant expression, and they gradually increase between E3.5 and E7.5 in PrE/VE, emVE and exVE; in contrast, S1P signaling-associated genes (synthesis, uptake, degradation and receptors) show variable expression kinetics, indicating interplay of several mechanisms of regulation (Kono et al., 2022). For instance, *Sphk2* and the S1P transporter *Spns1* (which is a putative S1P exporter from cells), show a kinetics somehow similar to that of *Plpp2/3*, while the expression of the intracellular S1P-degrading enzymes *Sgpl* and *Sgpp2* is somewhat maintained amongst the different stages or increases as development progresses, respectively. Notably, out of the five described S1P receptors, *S1pr2* is the only one with significant and increasing expression in VE lineages (Fig. S5). Although *Plpp3*, *Enpp2* and *Sphk1/2* KO mice share some phenotypic similarities, only the former show a subset of embryos with severe patterning defects, supporting that those could arise from simultaneous perturbations on LPA and S1P functions/signaling in VE (probably through increased LPA signaling but S1P receptor desensitization).

Exactly how the lack of PLPP3 prevents the downregulation of pluripotency factors in EB and during endodermal differentiation remains to be established. We speculate that simultaneous perturbations on LPA and S1P signaling could also be responsible for this phenotype due to their described roles in murine and human stem cells. However, we were not able to support this idea since transfection of wild-type *hPLPP3* was unable to downregulate NANOG expression in ‘differentiating’ *Plpp3*-deficient ESC. This could be explained by insufficient degradation of available lipids in the culture due to low transfection efficiency and low survival of cells transfected with *PLPP3*-overexpressing constructs. Alternatively, PLPP3 could be required in a cell-autonomous fashion for proper downregulation of pluripotency factors, for example, through its cell-to-cell adhesion domain or through its requirement in regulating intracellular lipid levels. Regarding the latter, one tempting hypothesis is related to the mitochondrial regulation of stem cell homeostasis. ESC and induced pluripotent stem cells, upon reprogramming, are more glycolytic, while differentiated cells are more dependent on oxidative phosphorylation. The metabolic state of stem cells produces epigenetic modifications that are important for stemness (Lisowski et al., 2018; Ryall et al., 2015). PLPP3 has been shown to regulate mitochondrial oxidative phosphorylation since PLPP3-deficient neonatal cardiomyocytes and their isolated mitochondria have reduced mitochondrial activity, increased basal superoxide production and increased glycolysis rates. LPA seems to have an involvement in this phenomenon, since treatment with this lipid augments basal superoxide production in wild-type cardiomyocytes but increases even more in PLPP3-deficient cardiomyocytes (Chandra et al., 2018). It has recently been described that treatment of hPSC with LPA produces great changes in metabolism and gene expression, including enhanced glycolysis and histone acetylation (Xu et al., 2021). Although it has not been studied if S1P could produce the same metabolic effects on ESC, participation of S1P in several aspects of mitochondrial function is well documented (Duan et al., 2022). It would be interesting to study the metabolic and epigenetic status of *Plpp3* mutant ESC and newly differentiating cells to establish if they are related to the alterations in downregulation of pluripotency associated genes and differentiation.

Our work has unveiled the participation of PLPP3 in regulating the YAP1-mediated signaling required for proper endoderm differentiation through the dephosphorylation of extracellular lipids, presumably LPA and S1P. Additionally, our study highlights a role for PLPP3 in regulating mESC exit from pluripotency by a mechanism that remains to be established. Our work could lay the groundwork for better understanding the role of lipid signaling-modulating enzymes in regulating stemness and differentiation during mouse early development.

## MATERIALS AND METHODS

### Derivation and culture of *Plpp3^F/F^* and *Plpp3*^Δ*F/*Δ*F*^ mouse ESC

mESC were cultured on a feeder layer of Mitomycin C (Sigma-Aldrich)-treated primary mouse embryonic fibroblasts (PMEF) in D15 [Dulbecco's modified Eagle medium (DMEM)-high glucose supplemented with 2 mM Glutamine, 50 U/ml penicillin, 50 μg/ml streptomycin, 1 mM sodium pyruvate and 0.1 mM nonessential amino acids (all reagents from Life Technologies), 0.1 mM β-mercaptoethanol (Sigma-Aldrich), and 15% ESC-tested FBS (Specialty Media, Millipore)].

All procedures used in this project were carried out according to protocol DEA141-18, approved by the Internal Committee for the Use and Care of Laboratory Animals (CICUAL) from the Institute of Cellular Physiology, Universidad Nacional Autónoma de México. For isolation of mESC, blastocysts from homozygous conditional allele 129.*Plpp3^tm3Stw/tm3Stw^* (referred to as *F/F* or *F*; Escalante-Alcalde et al., 2007) intercrosses were obtained in M2 medium [94.66 mM NaCl, 4.78 mM KCl, 1.71 mM CaCl_2_•2H_2_O, 1.19 mM KH_2_PO_4_, 1.19 mM MgSO_4_•7H_2_O, 4.15 mM NaHCO_3_, 20.85 mM HEPES, 23.28 mM sodium lactate, 0.33 mM sodium pyruvate, 5.55 mM glucose, 0.4% bovine serum albumin (BSA), 90 U/ml penicillin-G (potassium salt), 36 UI/ml streptomycin sulfate, 0.001% Phenol Red] and seeded in a 35 mm Petri dish with D15 with PMEF feeders. After 6 days, the ICM of each blastocyst was picked, trypsinized and transferred to a 96-well plate containing inactivated PMEF and D15 supplemented with 25 μM PD098059 (Sigma-Aldrich) and 3 μM GSK-3 Inhibitor IX (Calbiochem). The cells were expanded, tested for mycoplasma (Sigma-Aldrich) and genotyped as previously described (Escalante-Alcalde et al., 2007). ESC were karyotyped to evaluate chromosomal stability, and clones with at least 80% of normal karyotypes were selected for experimental procedures.

*F/F* ESC were transiently transfected with a *pCAG-Cre-Ires-eGFP* plasmid using Lipofectamine 2000 (Invitrogen). After 2 days, individual GFP^+^ clones were picked, trypsinized and transferred onto 96-well plates. GFP^–^ clones were isolated in the same experiment and used as control. The isolated clones were expanded and genotyped as previously described to select clones carrying the excised allele *Plpp3^tm3.1Stw/tm3.1Stw^* (referred to as Δ*F/*Δ*F* or Δ*F*; Escalante-Alcalde et al., 2007).

### EB differentiation

To evaluate size and morphology differences in EB, we performed the following experimental procedures. ESC were trypsinized, resuspended in D10 [DMEM-high glucose supplemented with 2 mM glutamine, 50 U/ml penicillin, 50 μg/ml streptomycin, 1 mM sodium pyruvate and 0.1 mM non-essential amino acids (all reagents from Life Technologies), 0.1 mM β-mercaptoethanol (Sigma-Aldrich), 10% FBS (Gibco)] and cultured for 1 h on a plate treated with gelatin 0.1% for removal of PMEF. Then, the cells were collected and counted, and a suspension of 12,000 cells/ml was prepared. Drops of 25 µl were placed on the upturned inner surface of a 100 mm bacteriological culture dish lid. The lid was placed on top of the dish containing 10 ml of PBS to prevent the drops from drying. The hanging drops were maintained for 2 days in these conditions, then EB were resuspended in 10 ml of D10 and transferred into a bacteriological dish. Medium was changed every other day.

For western blot and qPCR experiments, EB were generated in bulk, suspending 500,000 ESC feeder free in 10 ml of D10 in a bacteriological culture dish. The medium was changed every 2 days, and samples were taken on days 2, 4, 6 and 8 of differentiation.

### Endoderm differentiation protocol from ESC

We applied a protocol for directed endoderm differentiation in monolayer based on previous studies (Chen et al., 2013; Freyer et al., 2015; Yasunaga et al., 2005). We seeded 90,000 feeder-free ESC on round coverslips pretreated with 14 μg/ml of fibronectin in a four-well plate. The first day, cells were maintained in D0 [DMEM-high glucose supplemented with 2 mM Glutamine, 1 mM sodium pyruvate and 0.1 mM non-essential amino acids (all reagents from Life Technologies), 1% *N*-2 (Gibco), 2% B-27 (Gibco) and 2.5 μM Y27632 (Calbiochem)] to improve cell survival. Forty hours later, medium was replaced for D2 [DMEM-high glucose supplemented with 2 mM Glutamine, 1 mM sodium pyruvate and 0.1 mM nonessential amino acids (all reagents from Life Technologies) and 2% FBS (Gibco) and 50 ng/ml Activin A (Peprotech; for mesendoderm formation)]. On day 3, medium was exchanged for D3, consisting of D2 supplemented with 50 ng/ml Activin A (Peprotech) and 2 μM of the BMP-signaling inhibitor Dorsomorphin (Sigma-Aldrich) for anterior primitive streak/definite endoderm differentiation. Appropriate fresh medium was added to the cells daily. On days 4 and 5, differentiated cells were fixed with 4% PFA for immunofluorescence.

### Immunofluorescence

For ESC immunofluorescence, cells were seeded on coverslips treated with 0.1% gelatin, fixed with 4% paraformaldehyde for 15 min, rinsed with PBS for 5 min, and incubated in blocking solution (0.1% Triton X-100 and 5% BSA in PBS) for 1 h at room temperature. Then, cells were incubated with primary antibodies (Table S1) diluted in blocking solution for 1 h at room temperature or overnight at 4°C and then rinsed three times with PBS for 10 min, incubated with Alexa Fluor-conjugated secondary antibodies (Molecular Probes, Invitrogen) diluted 1:500 in blocking solution for 1 h at room temperature. The cells were rinsed three times with PBS and stained with a 1:1000 dilution of 1 mg/ml Hoechst for 15 min. Finally, the sample was rinsed and mounted using Vectashield (Vector Laboratories).

EB were fixed with 4% paraformaldehyde for 30 min and rinsed with PBS for 10 min. Then, EB were permeabilized 20 min with Triton X-100 0.25% (v/v) in PBS and blocked for 1 h with 5% BSA, 0.1% Tween in PBS. Primary antibodies (Table S1) were diluted in blocking solution, incubated for 2 h at room temperature or overnight at 4°C and rinsed with 0.1% Tween in PBS (PBST) for 10 min five times. Then, EB were incubated for 1 h with Alexa Fluor-conjugated secondary antibodies 1:500 in blocking solution and rinsed with PBST for 10 min five times. Finally, EB were incubated with 1:1000 1 mg/ml Hoechst solution for 30 min and rinsed with PBST five times. EB were mounted in slides using silicon separators with Vectashield.

### Fluorescence intensity quantification and cell counting

Images were acquired with a confocal microscope LSM800 (Zeiss). To analyze the frequency and intensity of ESC-stained nuclei, we used the Modular Interactive Nuclear Segmentation (MINS) program (Lou et al., 2014). To analyze intensities and morphological differences in differentiated cells, we used ImageJ (https://imagej.nih.gov/ij/index.html). During endodermal differentiation, the nuclei of the cells change in morphology to a more elongated shape, rendering cell detection by MINS more unreliable. Cell counting was performed using two different programs. In pluripotent culture conditions, we used the MINS program for nuclei detection, then we carried out fluorescence intensity quantification in each channel.

For endoderm differentiated cells, we used ImageJ for nuclear staining (FOXA2, NANOG, β-CATENIN, YAP1) and cytoplasmic quantifications (YAP1). For nuclear detections using ImageJ, TIFF files were converted to 16-bit format, background subtracted and image threshold adjusted for nuclei detection, then we converted this image to binary to select the regions of interest (ROI; individual nuclei). Using these ROI, green and red fluorescence were measured in the 16-bit format/background-subtracted files. Fluorescence was reported as the proportion of positive nuclei per analyzed image.

For the cytoplasmic detection of YAP1, we followed the same steps up to image conversion to binary in the green channel. Then, the image was inverted to select ROI in the cytoplasm. Using these ROI, green and red fluorescence were measured in the 16-bit format/background-subtracted files.

To obtain the YAP1 nuclear/cytoplasmic ratio per cell, three *z*-planes were selected per channel, and the corresponding quantifications were performed and averaged. Then, we divided the averaged fluorescence intensity between compartments. The average of the ratios in the total measured cells/image was reported.

To distinguish between populations with different levels of NANOG expression, we established a 28,000 arbitrary units threshold in order to distinguish populations with low and high fluorescence intensity. Data below 28,000 arbitrary units were considered low fluorescence intensity, and data above that threshold were considered high fluorescence intensity.

### Isolation of RNA, reverse transcription and qPCR

RNA of ESC and EB was isolated with TRIzol (Invitrogen). RNA was treated with recombinant DNAse I, RNAse-free (Roche) and was recovered using sodium acetate precipitation. cDNA synthesis was accomplished with a Transcriptor First Strand cDNA Synthesis Kit (Roche) with 2 µg of RNA. A dilution of 10 ng/μl of cDNA was used for qPCR; levels of gene expression were measured based on SYBR green detection with Step One Plus Real Time PCR (Applied Biosystems).

The PCR mix contained 1× SYBR green (Applied Biosystems), a final concentration of 300 nM of forward and reverse primers (Table S2) and 25 ng of cDNA in a volume of 12.5 μl. The program used in the detection was initial temperature 95°C for 2 min, then 95°C for 30 s and 60°C for 1 min for 40 cycles, and then the melt curve analysis was carried out with standard conditions.

### Western blot analysis

Feeder-free ESC and EB were homogenized in lysis buffer [50 mM Tris-HCl pH 8.0, 150 mM NaCl, 1% Igepal, Complete protease inhibitors 1X (Roche), 1 mM NaVO_4_ and 10 mM NaF]. Then, 40 μg of total protein was separated on 10% SDS-PAGE at 100 V for 180 min and transferred overnight at 30 V and 4°C to polyvinylidene fluoride membranes (Hybond-P, Amersham Pharmacia, GE Healthcare). Membranes were blocked for 1 h in milk or 5% albumin at room temperature and incubated with primary antibody (Table S3) in blocking solution overnight at 4°C, then three times rinsed and incubated with secondary antibody coupled to a peroxidase HRPT (Santa Cruz Biotechnology) for 1 h at room temperature. The membrane was developed using a luciferase-based reaction (ECL or Immobilon Millipore) and sensitive photographic film. Densitometric analysis was done using ImageJ software, and data were normalized against GAPDH and expressed as relative protein levels versus controls.

### Transfection experiments

For rescue experiments, we used the endoderm differentiation protocol described above. On day 2 of differentiation, cells were transfected with the corresponding plasmids (Table S4). Transfection was performed in 250 μl of OPTIMEM per well, containing Y27632 (ROCK inhibitor) at a final concentration of 2.5 μM, 2 μl Lipofectamine 2000 and plasmid at final concentration of 0.5 μg/250 μl. Lipofection was carried out for 4 h, the medium was exchanged for D2 plus Activin A, and the differentiation protocol was continued. Samples were analyzed on days 4 or 5 of differentiation. Using this protocol, we obtained a 20% transfection efficiency after 24 h; however, the amount of surviving transfected cells with any of the *Plpp3*-overexpressing plasmid versions decreased significantly.

### Statistical analysis

Experiments were independently repeated at least three times. Statistical analysis was performed using GraphPad Prism 5. In order to establish if data were parametric or non-parametric, we analyzed normal distribution and variance equality. For two group comparisons in qPCR, western blotting and proportion of FOXA2-positive cells, the Mann–Whitney test was used. For two group comparisons in nuclear/cytoplasmic ratio of YAP1, Student’ *t*-test was used. For *F/F* and Δ*F/*Δ*F* multiple group comparisons in qPCR, western blot, mean fluorescence intensity and marker-positive cells, the Kruskal–Wallis was applied. One-way ANOVA was performed in YAP1 nuclear/cytoplasmic ratio multiple comparisons. In transfection experiments, we compared all groups versus *F*-Empty. In all analyses, *P*<0.05 was considered statistically significant. Error bars correspond to the standard deviation.

### Visualization of scRNA-seq developmental gene expression data

The raw counts of scRNA-seq were obtained from the endoderm explorer webpage (Nowotschin et al., 2019; https://endoderm-explorer.com/) in plain text format. Data were filtered using ad hoc Python scripts to get the cell types and gene expression of interest. Finally, the heatmap was created plotting the mean expression of each gene in each cell type and developmental stage using the seaborn Python module (Waskom, 2021).

## Supplementary Material

10.1242/biolopen.059665_sup1 Supplementary information
