# Supplementary material for: Plpp3, a novel regulator of pluripotency exit and endodermal differentiation of mouse embryonic stem cells
Source: Biol Open. 2023 Jan 12;12(1):bio059665. doi: 10.1242/bio.059665 (PMC9867895; doi:10.1242/bio.059665)
Supplement: Supplementary information [file biolopen-12-059665-s1.pdf]

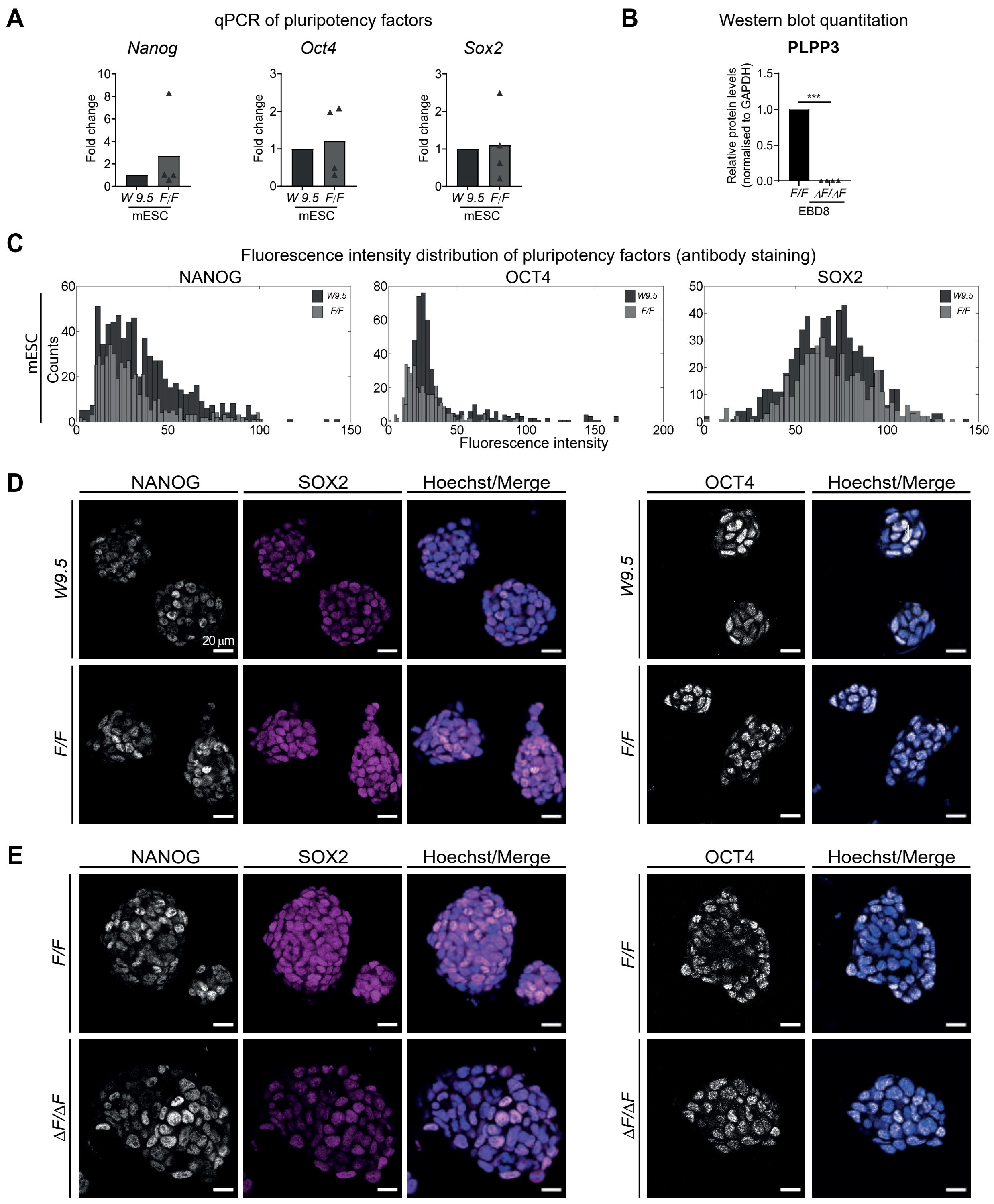


**Fig. S1. Characterization of pluripotency of *F/F* and Δ*F*/Δ*F* derived ES cell lines.** A) Fold change of pluripotency factors *Nanog*, *Oct4* and *Sox2* in *F/F* ES cells with respect to the W9.5 ES cells line (n=3). B) Bar plot of PLPP3 relative expression in *F/F* and Δ*F*/Δ*F* embryoid bodies differentiated 8 days (EBD8, n=3). n shows the number of independent experiments performed. Comparisons between *F/F* and Δ*F*/Δ*F* were made using the two-tailed Mann-Whitney test. *** p<0.001. C) Histograms showing distribution of nuclei fluorescence intensity of NANOG, OCT4 and SOX2 immunostainings in *F/F* and W9.5 ES cells. B) Representative images of immunostainings against NANOG, SOX2 and OCT4 in W9.5, *F/F* and Δ*F*/Δ*F* ES cells.

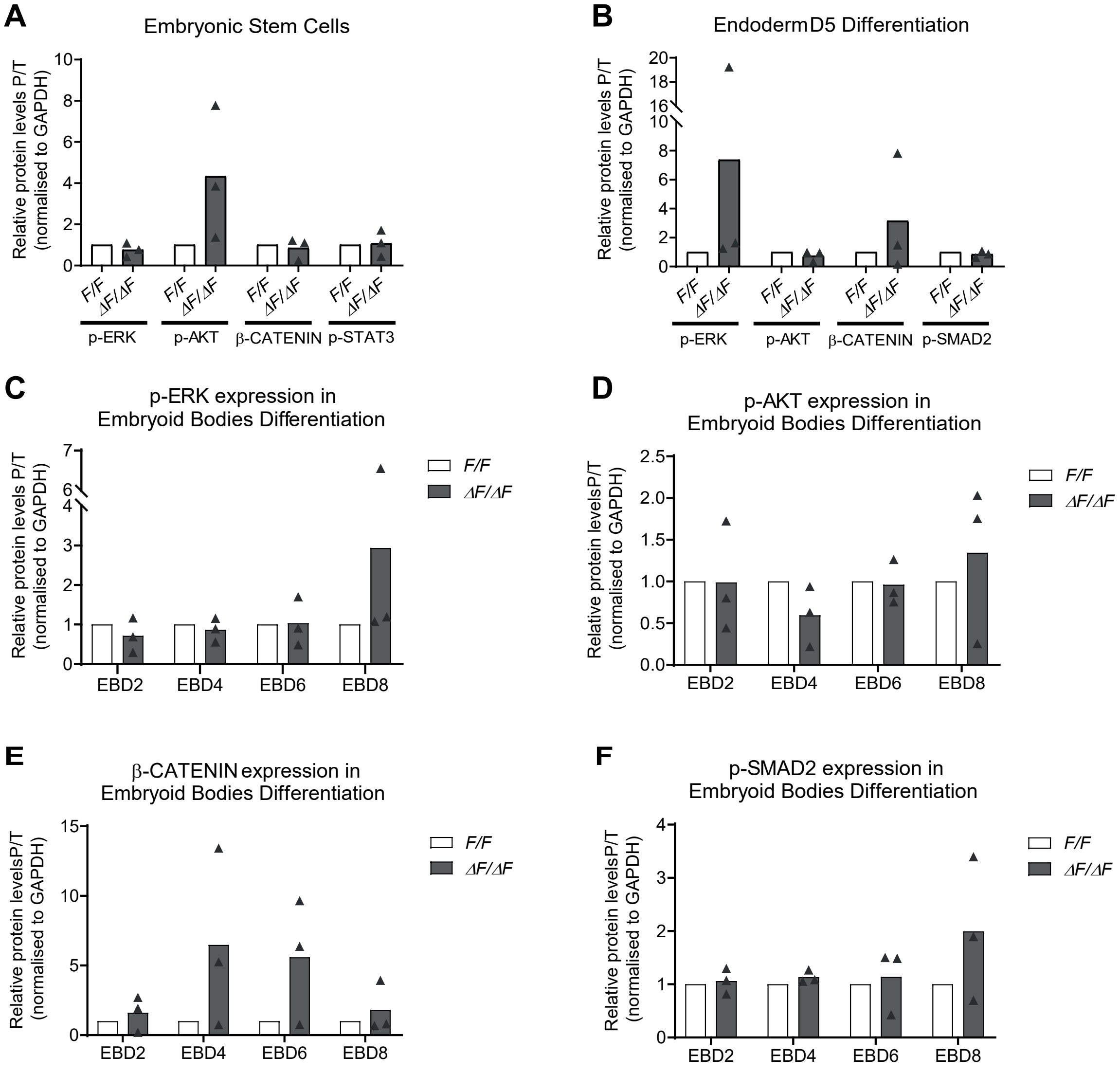


**Fig. S2. PLPP3 deficiency does not alter the activation of ERK1/2, AKT, β-CATENIN, STAT3 or SMAD2.** Graphs showing p-ERK, p-AKT, Active β-CAT-ENIN, p-STAT3 and p-SMAD2 normalized expression in *F/F* and Δ*F*/Δ*F* ES cells (A, n=3), ESC differentiated to endoderm D5 (B, n=3) and EB differentiated 2, 4, 6 and 8 days (C-F, n=3). Differentiation of several cell types in EB or different culture conditions could account for some of the differences observed in EB vs. endoderm differentiated cells. n shows the number of independent experiments performed. Comparisons were made using the Krus-kal-Wallis test.

Biology Open • Supplementary information

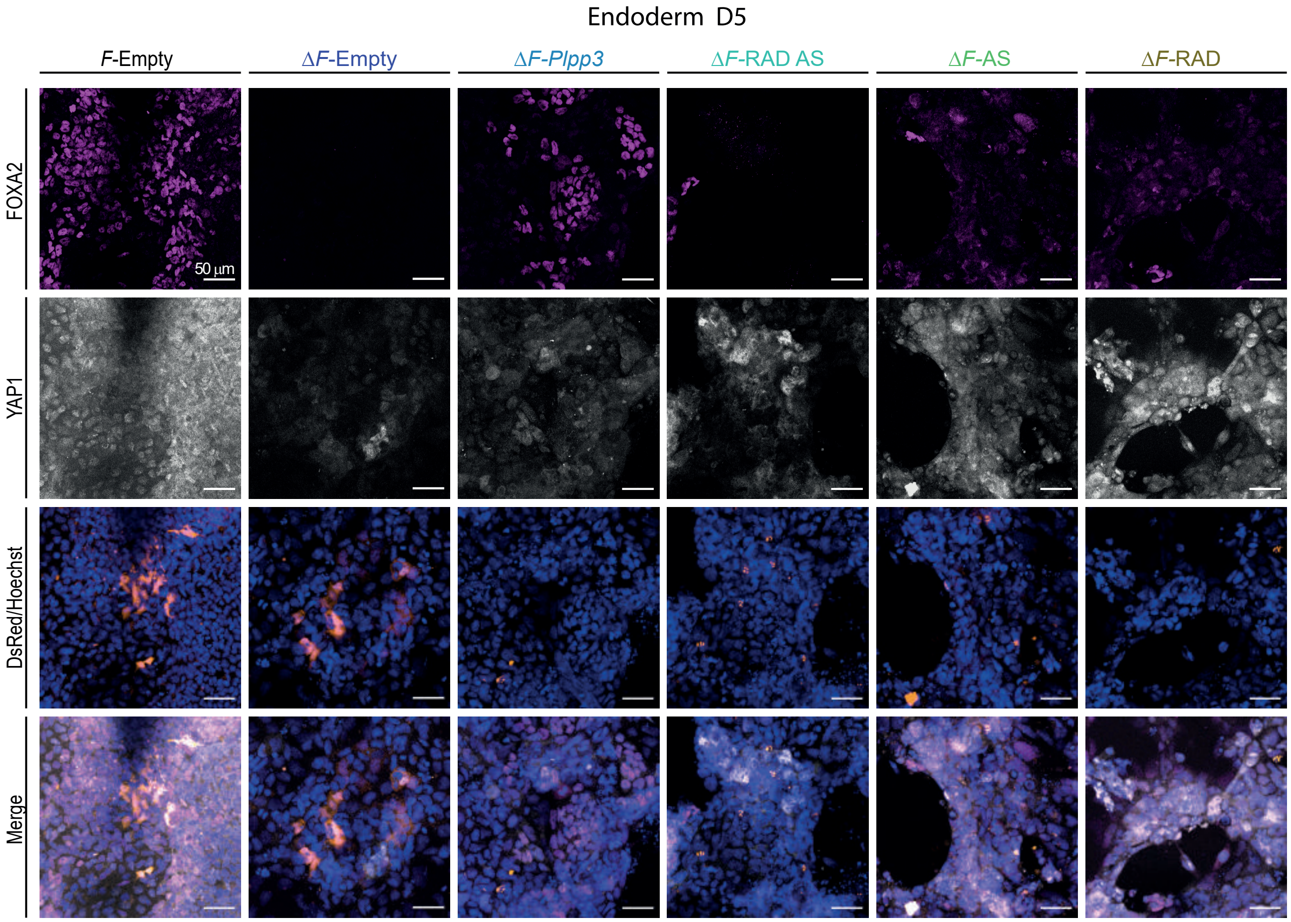


**Fig. S3. Immunostaining against FOXA2 and YAP1 in a representa-tive experiment evaluated on D5 of the endoderm directed differentiation.** Mutant cells transfected with the WT version of the enzyme express FOXA2 whilst when transfected with single domain or double domain mutants very few cells express this marker on D5. At this day, YAP1 distribution showed no significant differences between genotypes nor with any of the transfected versions of PLPP3. *F*, *F/F* cells; Δ*F*, Δ*F*/Δ*F* cells. Empty, *DsRed*-empty plas-mid; *Plpp3*, *DsRed*-wild-type *Plpp3*; RAD AS, *DsRed-Plpp3* integrin-binding motif and catalytic site double mutant; AS, *DsRed-Plpp3* catalytic site mutant; RAD, *DsRed-Plpp3* integrin-binding motif mutant.

Biology Open • Supplementary information

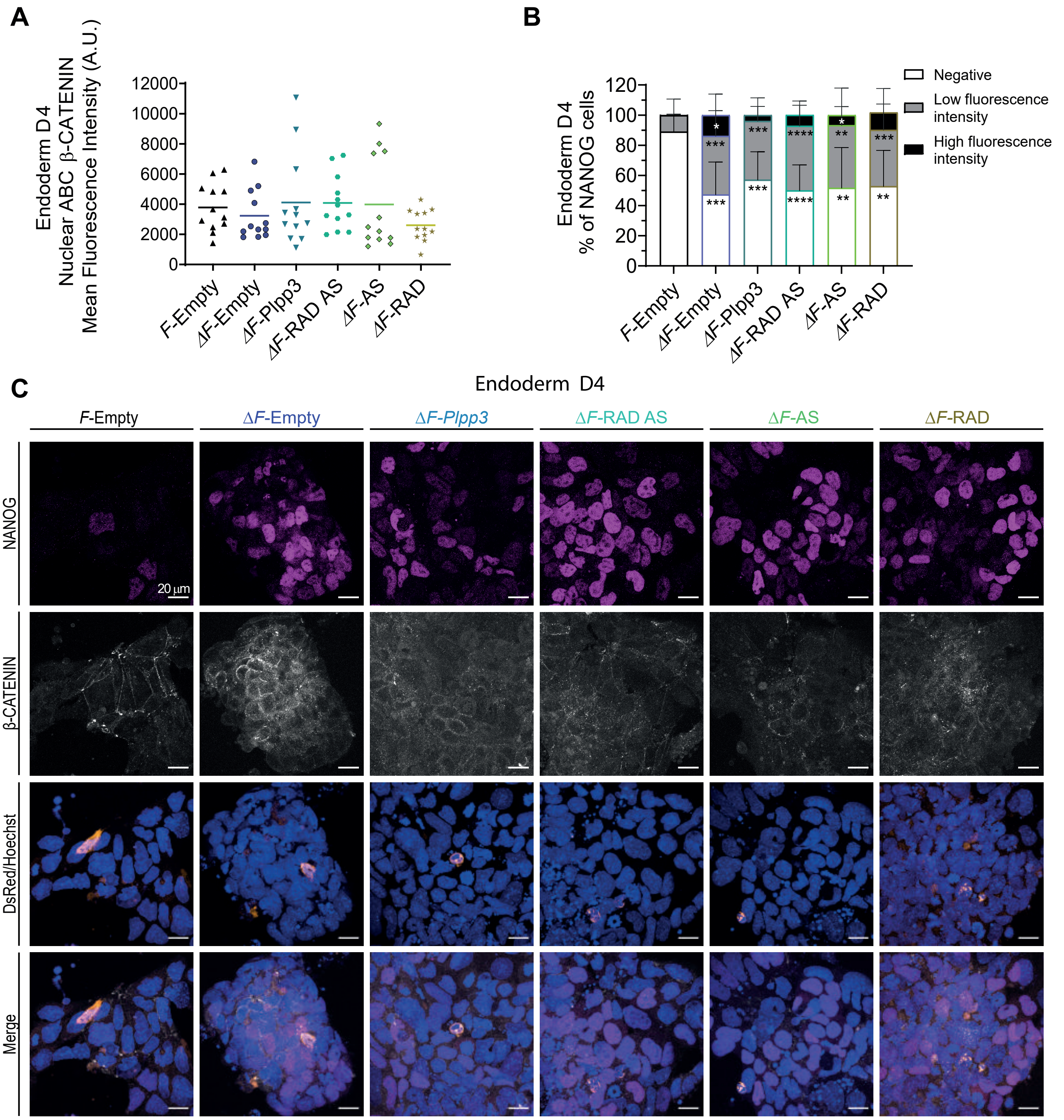


**Fig. S4. Quantification of NANOG fluorescence intensities and nuclear active β-CATENIN on D4 of endoderm directed differentiation** A) Mean fluo-rescence intensities of nuclear active β-CATENIN expression in cells transfected with the indicated vectors (n=3). B) Percentage of NANOG$^+$ nuclei with High and Low fluorescence intensity in cells transfected with the indicated vectors (n=3). n shows the number of inde-pendent experiments performed. Comparisons were made with respect to *F*-empty using the Kruskal-Wallis test. Data are shown as mean ± s.d. * p<0.05, ** p<0.01, *** p<0.001, **** p<0.0001. C) Active β-CATENIN and NANOG immunostaining evaluated on D4 of the endoderm directed differentiation. Representative experiment performed on cells transfect-ed with the indicated plasmids. *F*, *F/F* cells; Δ*F*, Δ*F*/Δ*F* cells.

Biology Open • Supplementary information

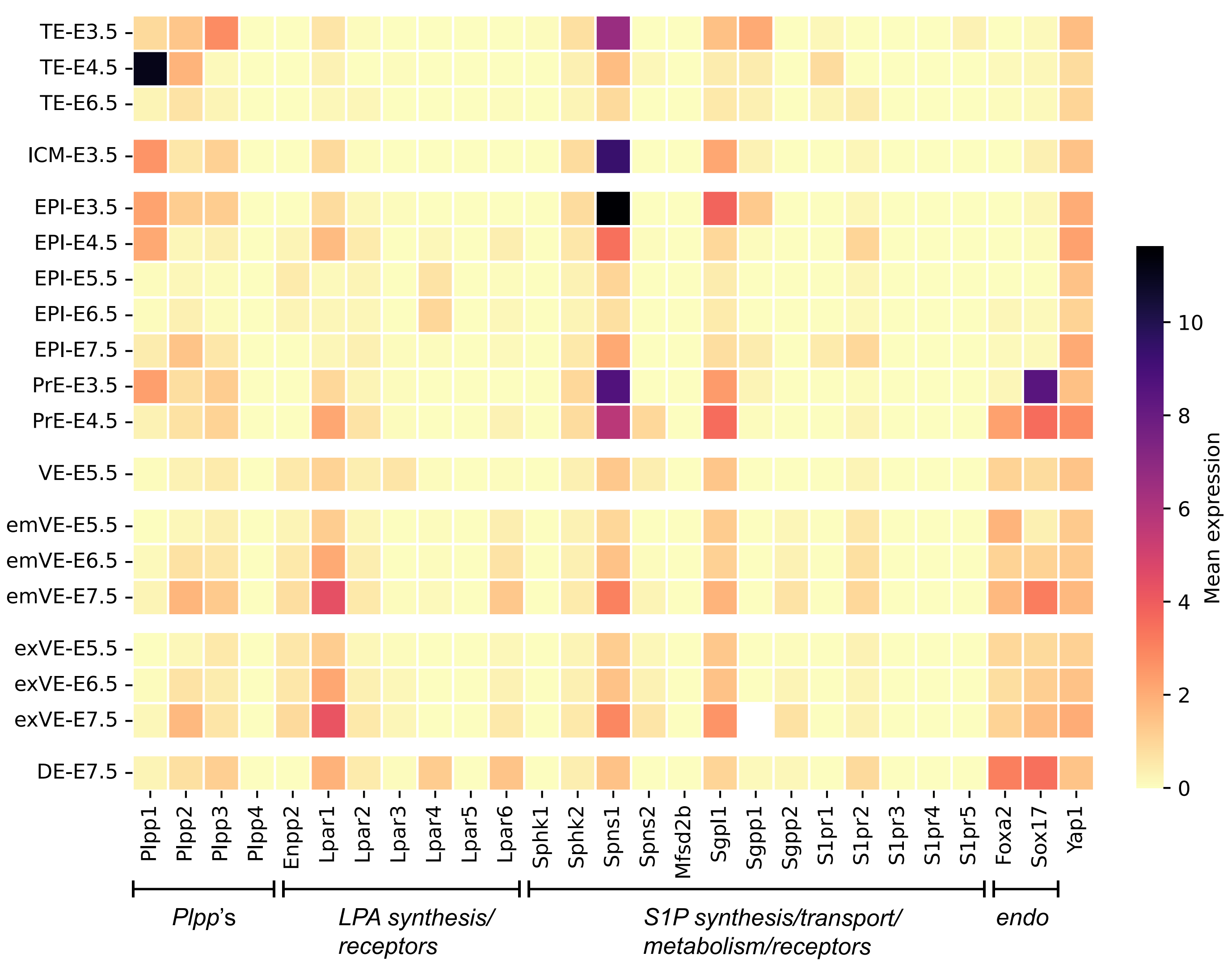


**Fig. S5. Gene expression analysis of the indicated genes in mouse embryos at E3.5-E7.5.** TE, trophectoderm; ICM, inner cells mass; EPI, epiblast cells; PrE, primitive endoderm; VE, visceral endoderm; emVE, embryonic visceral endoderm; exVE, extraembryonic visceral endo-derm; DE, definitive endoderm; endo, endoderm.

Biology Open • Supplementary information

**Table S1. List of antibodies used for immunofluorescence**

| Antibody | Dilution | Catalog |
|---|---|---|
| Anti-NANOG | 1:250 | eBioscience Cat. 14-5761 |
| Anti-OCT4 | 1:200 | BD Transduction Cat. 611202 |
| Anti-SOX2 | 1:200 | Millipore Cat. AB5603 |
| Anti-FOXA2 | 1:1000 | abcam Cat. Ab40874 |
| Anti-YAP1 | 1:200 | Santa Cruz Biotech. Cat. Sc-101199 |
| Anti-ABC β-catenin | 1:200 | Millipore Cat. 05-665 |

**Table S2. Primer sequences and fragment size produced by PCR reaction.**

| Primer | Sequence | Fragment size | Reference |
|---|---|---|---|
| Pluripotency | | | |
| *Nanog* Fw | 5'-TATCTGGTGAACGCATCTGG | 195 | Im *et al*, 2012 |
| *Nanog* Rv | 5'-GAAGTTATGGAGCGGAGCAG | | |
| *Oct4* Fw | 5'- TTGGGCTAGAGAAGGATGTGGTT | 217 | Chen *et al*, 2011 |
| *Oct4* Rv | 5'- GGAAAAGGGACTGAGTAGAGTGTGG | | |
| *Sox2* Fw | 5'-GCACATGAACGGCTGGAGCAACG | 207 | Chen *et al*, 2011 |
| *Sox2* Rv | 5'-TGCTGCGAGTAGGACATGCTGTAGG | | |
| Endoderm | | | |
| *Sox17* Fw | 5'- TATGGTGTGGG CCAAAGACGAA | 121 | Chew *et al*, 2011 |
| *Sox17* Rv | 5'- CCGCTTCTCTGCCAAG GTCAA | | |
| *Foxa2* Fw | 5'-CATCCGACTGGAGCAGCTA | 92 | Kim *et al*, 2010 |
| *Foxa2* Rv | 5'-TGTGTTCATGCCATTCATCC | | |
| *Gata4* Fw | 5'-TCTCACTATGGGCACAGCAG | 100 | Holtzinger *et al*, 2010 |
| *Gata4* Rv | 5'-GGGACAGCTTCAGAGCAGAC | | |
| *Gata6* Fw | 5'-TACACAAGCGACCACCTCAG | 110 | Yin *et al*, 2015 |
| *Gata6* Rv | 5'-TGTAGAGGCCGTCTTGACCT | | |
| *Afp* Fw | 5'- CCGGAAGCCACCGAGGAGGA | 234 | Clinkenbeard *et al*, 2012 |
| *Afp* Rv | 5'- TGGGACAGAGGCCGGAGCAG | | |
| Mesoderm | | | |
| *Brachyury* Fw | 5'-TTTCTTGCTGGACTTCGTGA | 196 | Im *et al*, 2012 |
| *Brachyury* Rv | 5'-TCCATTGAGCTTGTTGGTGA | | |
| *Mesp1* Fw | 5'- CGCCTGCCTACCCTAGACC | 150 | Klattenhoffet *et al*, 2013 |
| *Mesp1* Rv | 5'- AGGTTTCTAGAAGAGCCAGCA | | |
| *α-SMA* Fw | 5′-GTCCCAGACATCAGGGAGTAA | 102 | Veres-Székely *et al*, 2017 |
| *α-SMA* Rv | 5′-TCGGATACTTCAGCGTCAGGA | | |
| Ectoderm | | | |
| *Sox1* Fw | 5'- GCCGAGTGGAAGGTCATGTC | 97 | Hu *et al*, 2002 |
| *Sox1* Rv | 5'- TGTAATCCGGGTGTTCCTTCAT | | |
| Control | | | |
| *ß-Actin* Fw | 5'-TATTGGCAACGAGCGGTTCC | 138 | Oliver *et al*, 2010 |
| *ß-Actin* Rv | 5'-GCATAGAGGTCTTTACGGATGTC | | |

Biology Open • Supplementary information

**Table S3. List of antibodies employed in Western blot experiments.**

| Antibody | Dilution | Catalog |
|---|---|---|
| Anti-NANOG | 1:5000 | eBioscience Cat. 14-5761 |
| Anti-OCT4 | 1:500 | BD Transduction Cat. 611202 |
| Anti-pERK | 1:1000 | Cell signaling Cat. 9101 |
| Anti-ERK | 1:1000 | Cell signaling Cat. 9102 |
| Anti-pAKT | 1:2000 | Cell Signaling Cat. 4060 |
| Anti-AKT | 1:1000 | Cell Signaling Cat. 4691 |
| Anti-ABC β-catenin | 1:1000 | Millipore Cat. 05-665 |
| Anti-β-catenin | 1:1000 | Millipore Cat. 04958 |
| Anti-pSMAD2 | 1:1000 | Cell signaling Cat. 3108 |
| Anti-SMAD2/3 (Nodal) | 1:1000 | BD Transduction Labs. Cat. 610842 |
| Anti-pYAP1 | 1:1000 | Cell Signaling Cat. 4911 |
| Anti-YAP1 | 1:1000 | Santa Cruz Biotechnology Cat. Sc-101199 |
| Anti-PLPP3 | 1:3000 | Custom, Sigma-Aldrich, López-Juárez et al., 2011 |
| Anti-GAPDH | 1:5000 | Millipore Cat. MAB374 |
| Anti-STAT3 | 1:2000 | Cell Signaling Cat. 4904 |
| Anti-pSTAT3 | 1:1000 | Cell Signaling Cat. 9131 |

**Table S4. List of plasmids used for rescue experiments**

| Plasmid | Function |
|---|---|
| *DsRed-Empty* | Negative Control |
| *DsRed-Plpp3* | Wild type human *PLPP3* |
| *DsRed-RAD* | *PLPP3* w/integrin binding motif mutation * |
| *DsRed-AS* | *PLPP3* w/catalytic site mutation ** H251L |
| *DsRed-RAD AS* | *PLPP3* RAD and AS double mutant |

Constructs were PLPP3-DsRed fusions with the fluorescent protein fused in the PLPP3 C-terminus. *Humtsoe *et al*, 2005. **Zhang *et al*, 2000.

Biology Open • Supplementary information

**References Tables S2 and S4**

Chen T., Du J., Lu G. (2011). Cell growth arrest and apoptosis induced by Oct4 or Nanog knockdown in mouse embryonic stem cells: a possible role of Trp53. Mol Biol Rep 39(2):1855-61.

Chew L. J., Shen W., Ming X., Senatorov V. V., Chen H. L., Cheng Y., Hong E., Knoblach S., Gallo V. (2011). SRY-Box containing gene 17 regulates the Wnt/β-catenin signaling pathway in oligodendrocyte progenitor cells. J Neurosci; 31(39): 13921–13935.

Clinkenbeard E. L., Butler J. E., Spear B. T. (2012). Pericentral activity of alpha-fetoprotein enhancer 3 and glutamine synthetase upstream enhancer in the adult liver are regulated by β-catenin in mice. Hepatology 56 (5): 1892–1901.

Holtzinger A., Rosenfeld G. E., Evans T. (2010). Gata4 directs development of cardiac-inducing endoderm from ES cells. Developmental Biology 337: 63–73.

Hu Y., Ippolito J. E., Garabedian E. M., Humphrey P. A., Gordon J. I. (2002). Molecular characterization of a metastatic neuroendocrine cell cancer arising in the prostates of transgenic mice. J Biol Chem; 277(46): 44462-74. Epub 2002 Sep 11.

Humtsoe J.O., Bowling R.A. Jr., Feng S., Wary K.K. (2005). Murine lipid phosphate phosphohydrolase-3 acts as a cell-associated integrin ligand. Biochem Biophys Res Commun. Sep 30;335(3):906-19.

Im J. E., Song S. H., Kim J. Y., Kim K. L., Baek S. H., Lee D. R., Suh W. (2012). Vascular differentiation of multipotent spermatogonial stem cells derived from neonatal mouse testis. Experimental & Molecular Medicine; 44: 303-309.

Kim P. T. W., Hoffman B. G., Plesner A., Hegalson C. D., Verchere C. B., Chung S. W., Warnock G. L., Mui A. L. F., Ong C. J. (2010). Differentiation of Mouse Embryonic Stem Cells into Endoderm without Embryoid Body Formation. PLoS One 5(11): e14146.

Klattenhoff C. A., Scheuermann J. C., Surface L. E., Bradley R. K., Fields P. A., Steinhauser M. A., Ding H., Butty V. L., Torrey L., Haas S., Abo R., Tabebordbar M., Lee R. T ., Burge C. B .Boyer L. A. (2013). Braveheart, a Long Noncoding RNA Required for Cardiovascular Lineage Commitment. Cell 152 (3): 570–583.

Oliver T. G., Mercer K. L., Sayles L. C., Burke J. R., Mendus D., Lovejoy K. S., Cheng M. H., Subramanian A., Mu D., Powers S., Crowley D., Bronson R. T., Whittaker C. A., Bhutkar A., Lippard S. J., Golub T., Thomale J., Jacks T., Sweet-Cordero E. A. (2010). Chronic cisplatin treatment promotes enhanced damage repair and tumor progression in a mouse model of lung cancer. Genes & Development; 24: 837-852.

Veres-Székely A., Pap D., Sziksz E., Jávorsky E., Rokonay R., Lippai R., Kálmán T., Fekete A., Tulassay T., Szabó A. J., Vannay A. (2017). Selective measurement of α smooth muscle actin: why β-actin cannot be used as a housekeeping gene when tissue fibrosis occurs. BMC Mol Biol. 18: 12.

Yin N., Yao X., Qin Z., Wang Y., Faiola F. (2015). Assessment of Bisphenol A (BPA) neurotoxicity in vitro with mouse embryonic stem cells. Journal of Environmental Sciences 36: 181-187.

Zhang Q.X., Pilquil C.S., Dewald J., Berthiaume L.G., Brindley D.N. (2000). Identification of structurally important domains of lipid phosphate phosphatase-1: implications for its sites of action. Biochem J. Jan 15;345 Pt 2(Pt 2):181-4.

Biology Open • Supplementary information
